# *Dgcr8* deletion in the primitive heart uncovered novel microRNA regulating the balance of cardiac-vascular gene program

**DOI:** 10.1007/s13238-018-0572-1

**Published:** 2018-08-20

**Authors:** Xi Chen, Lin Wang, Rujin Huang, Hui Qiu, Peizhe Wang, Daren Wu, Yonglin Zhu, Jia Ming, Yangming Wang, Jianbin Wang, Jie Na

**Affiliations:** 1 Center for Stem Cell Biology and Regenerative Medicine, School of Medicine, Tsinghua University, Beijing, 100084 China; 2 Beijing Key Laboratory of Cardiometabolic Molecular Medicine, Institute of Molecular Medicine, Peking University, Beijing, 100871 China; 3 School of Life Sciences, Tsinghua University, Beijing, 100084 China

**Keywords:** microRNA, *Dgcr8*, Cardiovascular progenitor cells, miRNA-541, Single cell RNA sequencing

## Abstract

Primitive mammalian heart transforms from a single tube to a four-chambered muscular organ during a short developmental window. We found that knocking out global microRNA by deleting *Dgcr8* microprocessor in *Mesp1* cardiovascular progenitor cells lead to the formation of extremely dilated and enlarged heart due to defective cardiomyocyte (CM) differentiation. Transcriptome analysis revealed unusual upregulation of vascular gene expression in *Dgcr8* cKO hearts. Single cell RNA sequencing study further confirmed the increase of angiogenesis genes in single *Dgcr8* cKO CM. We also performed global microRNA profiling of E9.5 heart for the first time, and identified that miR-541 was transiently highly expressed in E9.5 hearts. Interestingly, introducing miR-541 back into microRNA-free CMs partially rescued their defects, downregulated angiogenesis genes and significantly upregulated cardiac genes. Moreover, miR-541 can target *Ctgf* and inhibit endothelial function. Our results suggest that microRNAs are required to suppress abnormal angiogenesis gene program to maintain CM differentiation.

**Electronic supplementary material:**

The online version of this article (10.1007/s13238-018-0572-1) contains supplementary material, which is available to authorized users.

## Introduction

The heart is one of the first functional organs to form during mammalian development and is composed of multiple cell types, including cardiomyocytes (CMs), endothelial cells (ECs), smooth muscle cells (SMCs) and mesenchymal cells (MCs) such as fibroblasts (Buckingham et al., 2005; Xin et al., 2013). The cardiac progenitor cells (CPCs) of the first and second heart field converge in the cardiac crescent in E7.5 mouse embryos. Fusion of the cardiac crescent at the midline formed the early cardiac tube at E8.0. The heart tube undergoes looping at E8.5, and the atria and ventricle become morphologically distinct at E9.5 (Buckingham et al., 2005; DeLaughter et al., 2016). Due to small size and *in vivo* development of the early mammalian embryo, the function of microRNAs during this important window was poorly understood. MicroRNAs (miRNAs) are small non-coding RNAs with an average length of ~22 nucleotides that negatively regulate the stability and translation of mRNA transcripts (Ambros, 2004; Lewis et al., 2005; Srivastava, 2006). During heart development, many miRNAs, such as miR-1 and miR-133, have been shown to control CM maturation and function (Heidersbach et al., 2013; Ivey et al., 2008; Liu and Olson, 2010). Despite their interesting functions, knocking-out individual miRNA in mice rarely caused lethality (Liu and Olson, 2010), and very few showed severe phenotype at early embryonic stages possibly due to that miRNAs often function redundantly and exist at saturating levels (Wang et al., 2008b).

Knocking-out key miRNA processing proteins such as DGCR8 has been used to study the functions of global miRNAs (Wang et al., 2007). The two double-stranded RNA binding domains (dsRBDs) of DGCR8 recognize primary miRNAs (pri-miRNAs) (Han et al., 2006), while the conserved C terminus interacts with Drosha to form the microprocessor. The pri-miRNAs were processed by microprocessor into short hairpins, named pre-miRNA, which subsequently exported into cytoplasm, and processed by Dicer into double-stranded mature miRNAs (Wang et al., 2007). *Dgcr8* conditional knock-out (cKO) in muscle cells lead to dilated cardiomyopathy and postnatal lethality, indicating that global miRNAs were essential for normal CM function (Rao et al., 2009). We reason that deletion of *Dgcr8* at the beginning of heart formation could reveal functions of global miRNAs during this important window of development, and provide a sensitive system to study the role of individual microRNA enriched in the early heart. Many microRNA loss-of-function studies conducted in embryo systems appeared to cause mild or even no phenotype, but careful study revealed increase in variation or reduced robustness of the biological process (Cassidy et al., 2016; Ebert and Sharp, 2012; Kasper et al., 2017). Recent advance in single cell RNA-sequencing technology makes it possible to measure global gene expression in every cell of an organ. This greatly facilitated the identification of the affected cell type by a gene mutation and the associated transcriptome changes (DeLaughter et al., 2016; Lescroart et al., 2018; Li et al., 2016; Liu et al., 2017; Zhou et al., 2016). In this study, we crossed mice carrying floxed *Dgcr8* alleles with transgenic mice in which the CRE recombinase was driven by early cardiovascular progenitor cell marker gene *Mesp1*. *Dgcr8* cKO embryos showed severe cardiac defect at E9.5. Global transcriptome and miRNA profiling revealed that without miRNAs, cardiac genes were downregulated but vascular genes were upregulated in the E9.5 hearts. Using single cell RNA-sequencing, we discovered significant upregulation of cell adhesion, glycolysis and angiogenesis genes that may explain the defect in cKO CMs. We identified that miR-541 was highly expressed in E9.5 hearts and was a strong repressor of angiogenesis. MiR-541 can also promote CM differentiation from pluripotent stem cells. These results provided new insights about the development of nascent myocardial cells *in vivo* and uncovered novel function of miRNA-541, that can potentially be useful to treat blood vessel hyperplasia diseases and pathological cardiac remodeling.

## Results

### *Dgcr8* deletion in *Mesp1* cardiovascular progenitor cells lead to severely dilated heart and embryonic lethality

**Figure 1:**
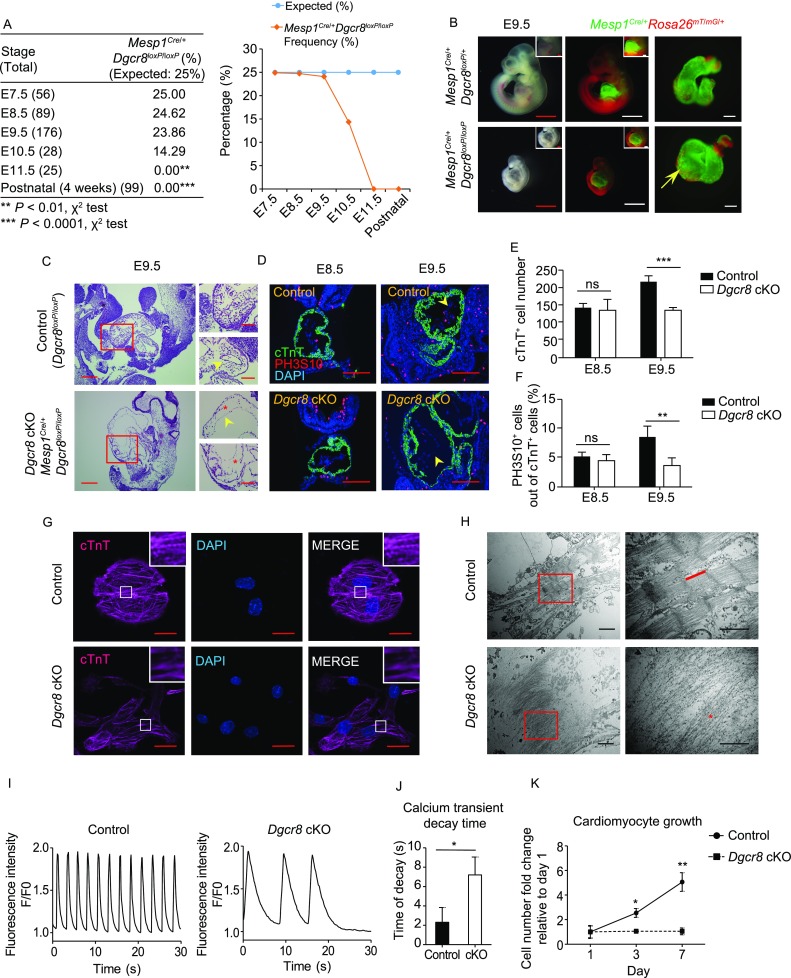
Deletion of *Dgcr8* in *Mesp1* progeny cells lead to embryonic lethality, heart dilation at E9.5 and defective CMs. (A) Number and percentage of *Mesp1*^*Cre*/+^; *Dgcr8*^*loxP*/*loxP*^ embryos recovered from E7.5 to postnatal stages. No *Mesp1*^*Cre*/+^*Dgcr8*^*loxP*/*loxP*^ embryos were recovered after E11.5. **P < 0.01, ***P < 0.0001. (B) E9.5 *Mesp1*^*Cre*/+^*Dgcr8*^*loxP*/+^; *Rosa26*^*mT*/*mG*/+^ (*n* = 42) and *Mesp1*^*Cre*/+^*Dgcr8*^*loxP*/*loxP*^; *Rosa26*^*mT*/*mG*/+^ (*n* = 53) embryos. Scale bar: 1 mm. Insert image scale bar: 200 μm. Right panels, dissected heart tubes, the arrow highlights the significant dilated heart of *Mesp1*^*Cre*/+^*Dgcr8*^*loxP*/*loxP*^ embryo. Scale bar: 100 μm. See also Movies S1 and S2. (C) H&E staining of E9.5 control (*Dgcr8*^*loxP*/*loxP*^) and *Dgcr8*-cKO (*Mesp1*^*Cre*/+^*Dgcr8*^*loxP*/*loxP*^) embryo sagittal sections. Stars indicate very thin ventricle walls. Arrow head indicated endocardium in the inner layer of the heart tube. Scale bar: 200 μm. (D) Immunostaining of cTnT (green) and PH3S10 (red) in E8.5 and E9.5 heart sections from control and *Mesp1*^*Cre/+*^; *Dgcr8*^*loxP/loxP*^ embryos. Arrow head indicated endocardium in the inner layer of the heart tube. DNA stained with DAPI (blue). Scale bar: 100 μm. (E) Quantification of cTnT^+^ cells on E8.5 and E9.5 heart sections from control and *Mesp1*^*Cre*/+^; *Dgcr8*^*loxP*/*loxP*^ embryos. Error bars represent s.e.m. (*n* = 3 embryos, 6 sections per embryo). Student’s unpaired t-test was used to determine statistical significance: ***P < 0.0001. (F) Quantification of PH3S10^+^/cTnT^+^ cell ratio on E8.5 and E9.5 heart sections from control and *Mesp1*^*Cre*/+^; *Dgcr8*^*loxP*/*loxP*^ embryos. Error bars represent s.e.m. (*n* = 3 embryos, 6 sections per embryo). Student’s unpaired *t*-test was used to determine statistical significance: **P < 0.01. (G) Immunofluorescence of sarcomere structure in dissociated CMs from E9.5 control and *Mesp1*^*Cre*/+^; *Dgcr8*^*loxP*/*loxP*^ embryos with cTnT antibody (pink). DAPI (blue) indicates nuclei. Scale bar: 50 μm. (H) TEM images showing sarcomere ultrastructure in CMs from E9.5 control and *Mesp1*^*Cre*/+^; *Dgcr8*^*loxP*/*loxP*^ embryos after 24 h culture *in vitro*. Distance between Z-lines is indicated with red lines. Disorganized sarcomeres are indicated by *. Scale bar: 2 μm. Insert image scale bar: 1 μm. (I) Quantification of Fluo-4 AM fluorescence intensity from control and *Mesp1*^*Cre*/+^; *Dgcr8*^*loxP*/*loxP*^ CMs isolated at E9.5. See also Movies S3 and S4. The fluorescence intensity of Ca^2+^ transients relative to diastolic fluorescence (F0) showed similar peak amplitudes but apparent differences in duration due to the slow contraction rhythm of the cKO CMs. (J) Mean durations of Ca^2+^-dependent Fluo-4 fluorescence transients plotted for control and *Mesp1*^*Cre*/+^; *Dgcr8*^*loxP*/*loxP*^ CMs. Data represent mean ± s.e.m. from three biological repeats. *P < 0.05. (K) Cell number of CMs from control and *Mesp1*^*Cre*/+^; *Dgcr8*^*loxP*/*loxP*^ embryonic hearts at day 1, 3 and 7 in the *in vitro* culture system. Data represent mean ± s.e.m. from three biological repeats. *P < 0.05, **P < 0.01, when compared to day 1 using Student’s unpaired *t*-test

*Mesp1* is the earliest cardiac progenitor marker (Bondue et al., 2011; Saga et al., 1999). We generated mice with *Mesp1* progeny cell-specific deletion of *Dgcr8* gene, by crossing *Mesp1*^*Cre*/+^ mice (Saga et al., 1999) with floxed *Dgcr8* mice (Wang et al., 2007). To monitor the *Dgcr8* cKO cells in the early embryo, we also generated *Dgcr8*^*loxP*/*loxP*^; *ROSA26*^*mTmG*/*mTmG*^ mice, upon crossing with *Mesp1*^*Cre*/+^*Dgcr8*^*loxP*/+^ mice, the CRE recombinase in *Mesp1* cells will excise the floxed tdTomato, thus permit the expression of EGFP in *Mesp1* progenies. In E7.5 embryos, both the cardiac crescent and yolk sac were strongly GFP positive. Subsequently, the GFP positive cells formed the heart tube. From E8.5, the entire heart tube was strongly GFP positive. By E10.5, the heart tube formed well-defined chambers (Buckingham et al., 2005), which consisted almost entirely of GFP cells (Fig. S1A and S1B). *Mesp1*^*Cre*/+^*Dgcr8*^*loxP*/*loxP*^ homozygous embryos survived at normal Mendelian ratios before E9.5, however, the number of embryos declined dramatically between E9.5 and E10.5, and no such embryos could be recovered beyond E11.5 (Fig. 1A). To find out the cause for embryonic lethality, *Mesp1*^*Cre*/+^*Dgcr8*^*loxP*/*loxP*^ embryos were obtained at different stages (Fig. S1B). At E9.5, *Mesp1*^*Cre*/+^*Dgcr8*^*loxP*/*loxP*^ embryos were much smaller in size, with markedly enlarged heart compared to *Mesp1*^*Cre*/+^*Dgcr8*^*loxp*/+^ embryos (Fig. 1B, Movies S1 and S2). Haematoxylin and eosin (HE) staining of embryo section revealed that the ventricular wall was significantly thinner in *Mesp1*^*Cre*/+^*Dgcr8*^*loxP*/*loxP*^ (hereafter referred to as *Dgcr8* cKO) embryos compared to control (*Dgcr8*^*loxP*/*loxP*^) embryos, the endocardium was present (Fig. 1C). Immunostaining showed that there was no significant difference in the number of Troponin T positive (cTnT^+^) cells between control and *Dgcr8* cKO embryos (Fig. 1D and 1E) in E8.5 heart tubes. However, by E9.5, the number of cTnT^+^ cell on the *Dgcr8* cKO heart section decreased 35.69%, and there were significantly fewer (~ 55.74%) dividing cells as marked by the Phospho-H3S10 staining (Fig. 1D and 1F).

To determine the cellular basis of the heart defect, E9.5 hearts from control and *Dgcr8* cKO embryos were dissected and dissociated into very small clusters, and cultured in a chemically defined medium, referred to as *in vitro* CMs culture medium (IVCC medium) supplemented with bFGF (4 ng/mL). Both control and *Dgcr8* cKO CMs attached well to the culture plate and kept beating after 24 h. Then they were fixed and stained for Troponin T (cTnT). Compared to the control CMs, *Dgcr8* cKO cells had poorly organized sarcomere structure (Fig. 1G). Transmission electron microscopy (TEM) images showed that *Dgcr8* null CMs contained only nascent myofibrils, while no clear Z-line could be observed. In contrast, mature Z lines and I bands were evident in control CMs (Fig. 1H). We also treated CMs with a Ca^2+^-sensitive dye, Fluo-4 AM to study the calcium handling ability of cKO cells. The rising slope and time to peak of Fluo-4 AM fluorescence were significantly different between the control and *Dgcr8* cKO CMs (Fig. 1I). Strong, rhythmic calcium influx was observed in control group (Fig. 1I and Movie S3). In contrast, in *Dgcr8* cKO group, the calcium transient had irregular rhythm, slower excitation cycle and longer decay time, suggesting decreased Ca^2+^ clearance from the cytosol and contractility (Fig. 1I, 1J and Movie S4). Finally, we analyzed proliferation ability of *Dgcr8* cKO CMs. Dissociated small cell clusters were cultured in IVCC medium with bFGF for 7 days. Cells were fixed and immunostained for cTnT on day 1, 3 and 7. CM numbers of each group were counted. By day 3 and day 7, the CM number in control group increased approximately 2.54 and 5.06 folds compared to day 1, respectively. While in *Dgcr8* cKO group, the cell number stayed the same after 7 days (Fig. 1K).

Taken together, above analysis clearly demonstrated that E9.5 *Dgcr8* cKO CMs had disrupted sarcomere structure, abnormal calcium transient and proliferation defect.

### Global gene expression profiling revealed that *Dgcr8* cKO embryonic heart cells had strong vascular signature

To find out the molecular mechanism underlying the defects in *Dgcr8* cKO embryonic heart, we isolated E8.5 and E9.5 hearts from control (*Dgcr8*^*loxP*^^/^^*loxP*^; *Rosa26*^*mT*/*mG*/+^) and *Dgcr8* cKO (*Mesp1*^*Cre*/+^*Dgcr8*^*loxP*/*loxP*^; *Rosa26*^*mT*/*mG*/+^) embryos, performed high-throughput RNA-sequencing and analyzed the changes in global gene expression. 810 genes were significantly differentially expressed (41 up and 769 down) in the E8.5 cKO heart tube compared with control heart tube (Fig. S2A). Q-PCR results showed that the levels of *Dgcr8* and key cardiac genes *Gata4*, *Nkx2–5*, *Tnni3* have not been significantly downregulated at this stage, but the expression of *Cxcr4* and *Pdgfra,* which are markers of mesoderm progenitor cells, were elevated (Fig. S2B). The presence of *Dgcr8* transcripts in E8.5 hearts suggested that there will be remaining miRNAs (Fig. S2B), which explained the lack of obvious phenotype and small change in gene expression at this stage.

**Figure 2:**
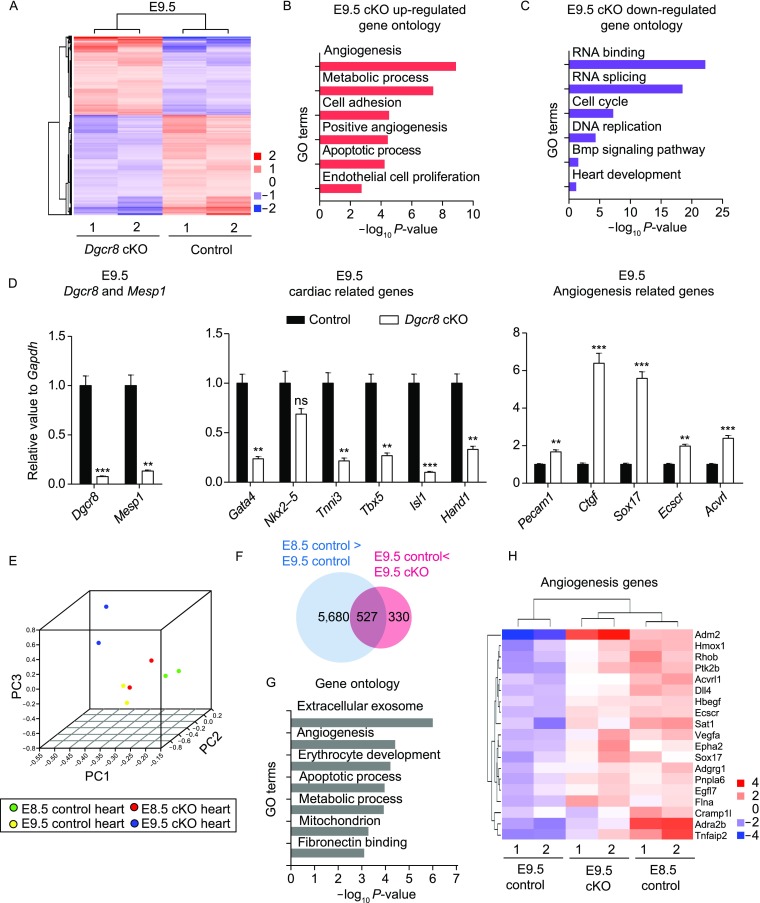
RNA high-throughput sequencing analysis of control and *Dgcr8* cKO mouse embryonic hearts at E8.5 and E9.5. (A) Heatmap showing differentially expressed genes between control (*Dgcr8*^*loxP*/*loxP*^; *Rosa26*^*mT*/*mG*/+^) and *Dgcr8* cKO (*Mesp1*^*Cre*/+^*Dgcr8*^*loxP*/*loxP*^; *Rosa26*^*mT*/*mG*/+^) hearts at E9.5. Values represent normalized mean centered log_2_ of FPKM for each sample. Higher expressed genes in red, lower expressed genes in blue (*n* = 2). (B and C) GO analysis of upregulated and downregulated genes in E9.5 *Dgcr8* cKO hearts. (D) Q-PCR quantification of expression level of *Dgcr8* and *Mesp1*, cardiac marker genes, and angiogenesis genes in E9.5 embryonic hearts. Data represent mean ± s.e.m. from three biological repeats. Student’s *t* test was used to determine statistical significance: **P < 0.01, ***P < 0.0001. (E) Principle component analysis (PCA) of genes expressed in E8.5 and E9.5 control and *Dgcr8* cKO hearts. (F) Venn diagram showing 527 genes were downregulated from E8.5 to E9.5 in the heart, but became upregulated in E9.5 *Dgcr8* cKO hearts compared with control hearts. (G) Gene Ontology analysis of 527 overlapped genes in (F). (H) Expression heatmap of angiogenesis genes that downregulated from E8.5 to E9.5 in the control hearts, but upregulated in E9.5 *Dgcr8* cKO hearts compared with E9.5 control. Values represent normalized mean centered log_2_ of FPKM for each sample

By E9.5, 857 genes were upregulated and 1,080 genes were downregulated in the *Dgcr8* cKO hearts (Fig. 2A). Interestingly, gene ontology (GO) enrichment analysis showed that genes involved in angiogenesis, metabolic process and apoptosis were upregulated (Fig. 2B and Table S2A), meanwhile, genes involved in RNA splicing, cell cycle, DNA replication, BMP4 signaling and heart development were downregulated (Fig. 2C and Table S2B). Heatmaps of genes in significantly up or downregulated GO class were listed in Fig. S2C. At E9.5, *Dgcr8* transcript level decreased markedly (Fig. 2C). The downregulation of cardiac genes, *Gata4*, *Tnni3*, *Tbx5*, *Isl1*, *Hand1*, and up-regulation of angiogenesis genes, *Pecam1*, *Ctgf*, *Sox17*, *Ecscr*, *Acvrl*, were also confirmed by q-PCR (Fig. 2D). Principle component analysis (PCA) showed that the transcriptome of E9.5 control and *Dgcr8* cKO hearts separated more far apart than that of E8.5 control and *Dgcr8* cKO hearts (Fig. 2E). Similar to the results of GO analysis, gene set enrichment analysis (GSEA) coupled with the pathway gene set data of Kyoto encyclopedia of genes and genomes (KEGG) showed enhanced gene expression related to vascular smooth muscle contraction and glycolysis in *Dgcr8* cKO group, while genes involved in spliceosome and DNA replication pathways decreased (*P* < 0.05, FDR < 0.25; Fig. S2D, Tables S4A and S4B). These results suggest that miRNA played important roles in vascular gene program repression.

In the control hearts, 6,207 genes were downregulated at least 1.5-fold from E8.5 to E9.5. Among them, 527 genes were upregulated more than 1.5-fold in E9.5 *Dgcr8* cKO hearts. These genes are likely to be repressed by miRNAs (Fig. 2F). The top GO categories associated with these 527 genes including angiogenesis and erythrocyte development, which were related to vascular development (Fig. 2G and Table S2C). The expression of several well-known positive regulators of angiogenesis such as *Egfl7*, *Sox17*, *Vegfα* and *Ctgf*, were significantly upregulated in E9.5 *Dgcr8* cKO hearts, indicating that they were highly likely to be targets of miRNAs at this stage (Fig. 2H). Sum together, the abnormal transcriptome of E9.5 *Dgcr8* cKO hearts was in accordance with the phenotype of the severely dilated and dysfunctional mutant heart.

### Single cell analysis of Dgcr8 cKO CMs revealed derepression of angiogenesis genes

The upregulation of angiogenesis genes in E9.5 *Dgcr8* cKO hearts may attribute to two reasons: the derepression of vascular related genes in *Dgcr8* cKO CMs, or the increase of EC proportion in the cKO heart. To distinguish between these two possibilities, we performed single cell RNA-seq of cKO and control hearts. We used homozygous *Dgcr8*^*loxp*/*loxp*^*; ROSA26*^*mT*/*mG*/+^, without any Cre alleles as control. The tdTomato fluorescence of the control group could be a marker to estimate the single cell sequencing quality in the following step, and the phenotypes of the control mice were normal from embryonic stages to adult.

**Figure 3:**
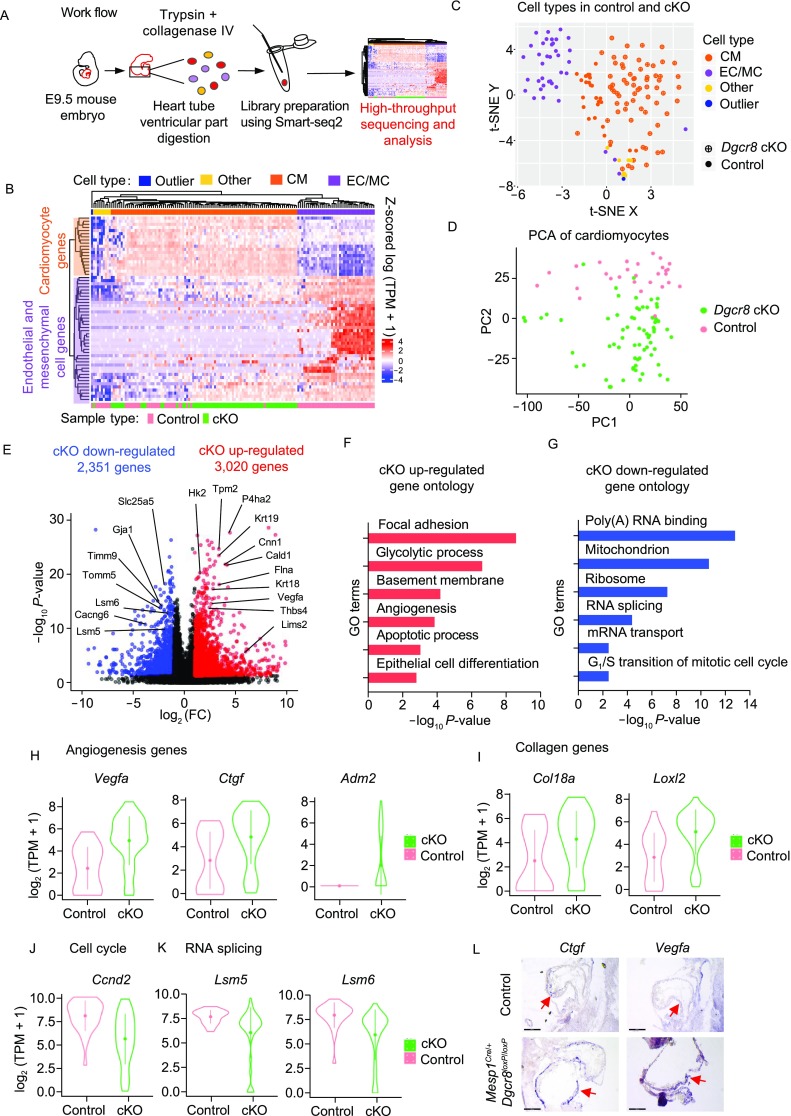
Single-cell high-throughput sequencing analysis of E9.5 control and Dgcr8 cKO Heart. (A) Workflow for single cell RNA sequencing experiment. (B) Hierarchical clustering identified different cell types in cKO (*Mesp1*^*Cre*/+^*Dgcr8* ^*loxP*/*loxP*^; *Rosa26*^*mT*/*mG*/+^) and control (*Dgcr8*^*loxP*/*loxP*^; *Rosa26*^*mT*/*mG*/+^) single cells. CM, Cardiomyocyte; EC/MC, Endothelial/mesenchymal. (C) t-SNE plots of all cells in cKO and control group. Color and shape labeling indicate assigned cell type for each single cell. (D) PCA graph of 96 single CMs (28 control cells and 68 cKO cells). (E) Volcano plot showing genes significantly up and downregulated in cKO single CMs compared to control CMs. Fold-change (cKO/control) were calculated using mean expression level of genes in each genotype. *P*-values were obtained by Mann-Whitney-Wilcoxon rank-sum test. Red dots represented significantly upregulated genes (*P* < 0.05 and log_2_(fold-change) > 0.6), while blue dots represented significantly downregulated genes (*P* < 0.05 and log_2_(fold-change) < − 0.6). (F and G) GO analysis of significantly upregulated and downregulated genes in E9.5 cKO single CMs compared to control CMs. (H–K) Violin plot showing the expression levels of representative genes in important GOs, angiogenesis genes (H), collagen genes (I), cell cycle genes (J), and RNA splicing genes (K), x axis: control or cKO group, y axis: log_2_(TPM + 1). (L) RNA *In situ* hybridization of angiogenesis genes *Vegfa* and *Ctgf* in NC and cKO hearts. Arrow highlights gene expression. Scale bar: 500 μm

The ventricular part of control and cKO E9.5 hearts were dissected and digested into single cell suspension. 96 single cells of each genotype were manually picked, followed by RNA-seq library construction and high-throughput sequencing (Fig. 3A). We obtained 76 high-quality single cell libraries from control and cKO ventricles respectively and there was no batch effect between two sequencing experiments (Fig. S3A, S3C and Table S3A). To determine whether the cells were progeny of Mesp1 in the cKO group, we included GFP and tdTomato sequences in the reference genome for mapping and expression level quantification (Showell and Conlon, 2007). Among 76 cKO single cells, high GFP and low tdTomato cells were destined to be Mesp1 progeny cells, while 7 low GFP and high tdTomato cells were considered to be control cells that had not expressed Mesp1-Cre. In 76 control single cells, 2 high GFP and low tdTomato cells were considered not to be control cells. These 9 cells with abnormal reporter gene expression were excluded from the following comparative analysis (Fig. S3B and Table S3A).

We first separated CM, EC/MC and other cell types based on lineage markers used in published studies (Li et al., 2016). 28 cells from the control group and 68 cells from the cKO group were characterized to be CMs (Fig. 3B and 3C and Table S3B). Heatmap and PCA graph showed that most single cKO CMs could be clearly separated from control CMs (Fig. 3D). Volcano plot revealed that 3,020 and 2,351 genes were significantly up and downregulated in single cKO CM cells compared with single control CMs (Fig. 3E), based on the Mann-Whitney-Wilcoxon rank-sum test (Table S3C). Among them, genes that were also differentially expressed in bulk RNA sequencing (FC < 1.5, *P* < 0.05) were listed in Table S3F. Upregulated genes in single cKO CMs were enriched in GO class of focal adhesion, glycolysis process, angiogenesis and epithelial cell differentiation (Fig. 3F, Table S3D and S3G). Notably, 15 angiogenesis genes were in this class, including *Anxa2*, *Ctgf*, *Pecam1*, *Vegfa*, *Adm2*, *Flna*, *Hspg2*, *Rhob*, etc. (Fig. S3D and Table S3I). Downregulated genes were predominantly classified in GO class of polyA RNA binding, mitochondrion, ribosome and RNA splicing (Figs. 3G, S3D, Table S3E, S3H and S3J). The GO enrichment analysis of differentially expressed genes in single CMs was similar with those in the bulk RNA-seq data (Fig. 2B and 2C). We selected representative genes from each GO class to shown as violin plots (Fig. 3H and 3I). For example, *Vegfa* and *Ctgf* were important angiogenesis genes normally lowly expressed in CMs but enriched in ECs and mesenchymal cells, their expression level increased significantly (Fig. 3H). RNA *in situ* hybridization also confirmed their upregulation in the cKO heart (Fig. 3L). *Adm2* was not detectable in ventricular control CMs, but highly expressed in cKO CMs (Fig. 3H). Sum above, our single cell RNA-seq analysis clearly demonstrated that the profound dysregulation of gene expression in *Dgcr8* cKO CMs could well be the principle cause of the dramatically dilated heart tube, and the abnormal increase of angiogenesis genes reflected a dramatic shift in gene expression program in microRNA-free CMs.

### MiRNA profiling of E9.5 embryonic heart

**Figure 4:**
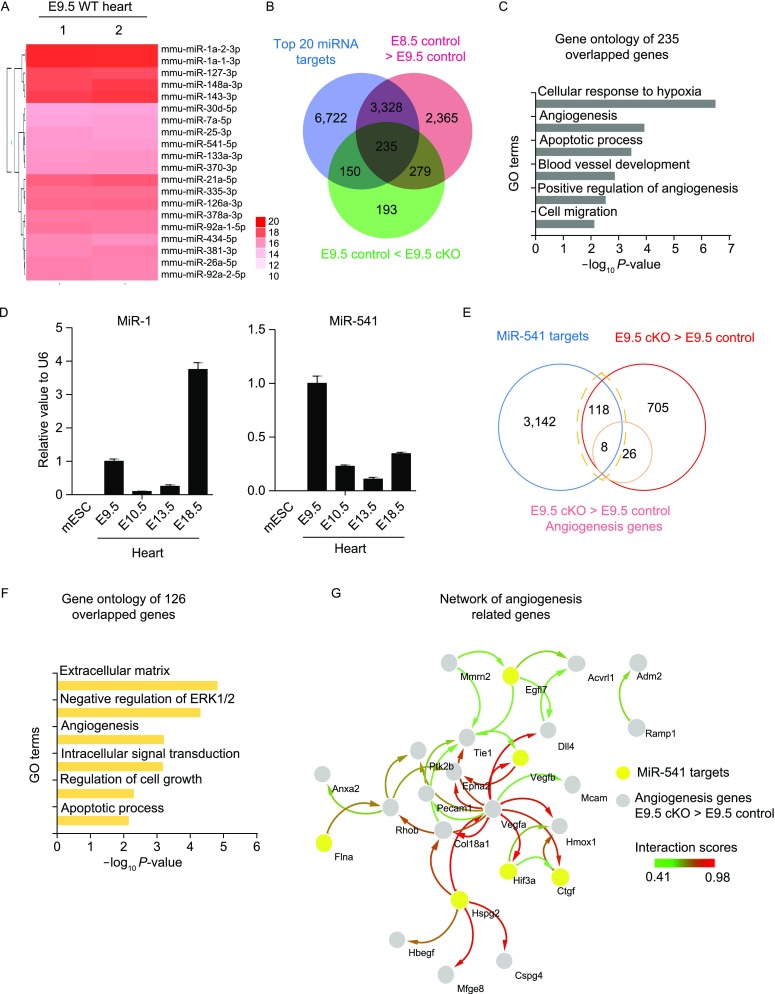
Global miRNA profiling in E9.5 mouse embryonic heart. (A) Heatmap showing the top 20 most highly expressed miRNAs in E9.5 WT embryonic heart. Values represent log_2_ of counts for each sample. (B) Venn diagram depicting the number of putative target genes of the top 20 miRNAs predicted by TargetScan (blue), genes downregulated from E8.5 to E9.5 in the control hearts (red) and genes upregulated in E9.5 *Dgcr8* cKO heart at E9.5 (green), and overlapped genes between these groups. (C) GO analysis of 235 overlapped genes among three groups in (B). (D) Q-PCR confirmation of miRNA-1 and miRNA-541 expression in embryonic heart at different stages and in mESCs. Data represent mean ± s.e.m. from three biological repeats. (E) Venn diagram depicting number of putative target genes of miRNA-541 predicted by TargetScan that overlapped with genes upregulated in E9.5 *Dgcr8* cKO hearts. (F) GO analysis of 126 overlapped genes in (E). (G) Cytoscape network generated from 34 angiogenesis genes that upregulated in E9.5 *Dgcr8* cKO heart, including 8 putative miR-541 targets. Node color represents two types of genes, yellow and grey represents miR-541 targets and non-miR-541 targets respectively. Edge is weighted by combined interaction scores, high values correspond to red color whereas low values to green color

To discover miRNAs might be responsible for the phenotype of *Dgcr8* cKO hearts, we performed miRNA sequencing of E9.5 wildtype (WT) hearts. The top 20 highest expressed miRNAs were shown in the heatmap graph (Fig. 4A), and their expression counts were listed in Table S4. Our results showed that miR-1 was the most abundant miRNA (20.45%) in E9.5 hearts, although not quite as high as its content in the adult heart (nearly 40%) (Rao et al., 2009). Many of the top 20 miRNAs were also reported to be enriched in the adult heart, such as miR-378a, miR-26a, miR-133a and miR-30 family. MiR-126, a miRNA highly expressed by ECs (Fish et al., 2008; Wang et al., 2008a), was also detected, possibly due to the ECs in the embryonic heart. We predicted targets of the top 20 miRNAs using TargetScan (Lewis et al., 2003). Among the 10,435 putative targets, 235 genes decreased more than 1.5 folds from E8.5 to E9.5 in control hearts but upregulated at least 1.5 folds in E9.5 *Dgcr8* cKO heart (Fig. 4B). GO analysis indicated that these genes were predominantly involved in angiogenesis and blood vessel morphogenesis (Fig. 4C and Table S2D). We speculate that the upregulation of this group of genes in E9.5 *Dgcr8* cKO hearts was due to lack of miRNAs inhibiting endothelial program, and subsequently contributed to the extremely dilated heart phenotype.

To identify such miRNA, we performed a series of experiments. MiR-541-5p, which was among the top 20 most abundant miRNAs in E9.5 hearts, displayed interesting dynamic expression pattern during heart development. Its level was the highest in E9.5 hearts, then decreased in E10.5, E13.5 and E18.5 hearts (Fig. 4D). This was different from miR-1a-3p, whose level was higher in later embryonic stages (Fig. 4D). Both miR-1a-3p and miR-541-5p were not detectable in undifferentiated mouse embryonic stem cells (Fig. 4D). We predicted the target genes of miR-541-5p using TargetScan (Lewis et al., 2003). 126 of its targets overlapped with genes upregulated in E9.5 *Dgcr8* cKO hearts, and 6 of them, including *Egfl7*, *Ctgf*, *Vegfb*, *Hif3a*, *Flna* and *Hspg2*, were key regulators of blood vessel development (Fig. 4E,4F and Table S2E). Moreover, network analysis with Cytoscape (http://cytoscape.org/) revealed that they were connected in a protein interactive network (Fig. 4G). Taken together, the transient upregulation of miR-541-5p in E9.5 hearts strongly suggests that it may function to suppress vascular gene program during this specific time window.

### MiR-541 partially rescued differentiation defects in *Dgcr8* cKO CMs

**Figure 5:**
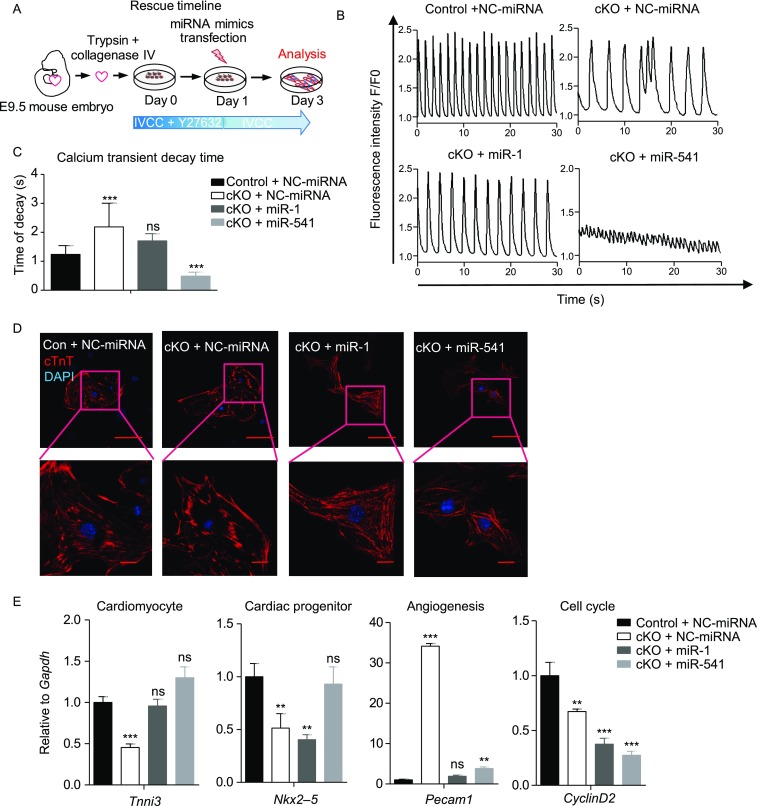
**MiR-1 and miR-541 rescued the defects of** ***Dgcr8*** **cKO CMs in an** ***in vitro*** **culture system**. (A) Schematic view of E9.5 CMs *in vitro* culture and miRNA transfection. (B) Quantification of Fluo-4 AM fluorescence intensity of control (*Dgcr8*^*loxP*/*loxP*^) and *Dgcr8* cKO CMs 48 h after NC-miRNA, miR-1 or miR-541 transfection. See also Movies S5 and S6. (C) Mean durations of Ca^2+^-dependent Fluo-4 fluorescence transients plotted for control and cKO CMs 48 h after transfection with NC-miRNA, miR-1 or miR-541. Data represent mean ± s.e.m. from three biological repeats. ***P < 0.0001 when compared to control + NC-miRNA using Student’s unpaired *t*-test. (D) Immunostaining of cTnT showing sarcomere structure in control and *Dgcr8* cKO CMs 48 h after transfection with NC-miRNA, miR-1, or miR-541 mimics, cTnT (pink), DAPI (blue) indicates nuclei. Scale bar: 50 μm. Insert image scale bar: 10 μm. (E) Q-PCR analysis of gene expression in control and *Dgcr8* cKO CMs after transfection with NC-miRNA, miR-1, or miR-541 and culturing for 48 h. Mean ± s.e.m. from three biological repeats are shown. **P < 0.01, ***P < 0.0001 when compared to control + NC-miRNA using Student’s unpaired *t*-test

As *Dgcr8* cKO CMs do not have miRNAs, they provide a baseline system to uncover the function of specific miRNAs after introducing back. MiR-1 was previously reported to play critical roles in promoting cardiac muscle cell maturation (Heidersbach et al., 2013), and here it was used as the positive control to evaluate the function of miR-541. We dissociated E9.5 hearts into very small cluster of cells and cultured them in IVCC medium. 24 h after seeding, cells were transfected with miRNA mimics: negative control miRNA (NC-miRNA), miR-1-3p and miR-541-5p (hereafter referred to as miR-1 and miR-541). 48 h after transfection, cells were collected for immunostaining, calcium transient imaging and gene expression analysis (Fig. 5A). The NC-miRNA did not affect the calcium transient in control and *Dgcr8* cKO cells, while miR-1 transfected *Dgcr8* cKO CMs restored rhythmic calcium influx and the time of decay to the level similar to control cells transfected with NC-miRNA (Fig. 5B and 5C). Interestingly, miR-541 transfected *Dgcr8* cKO CMs significantly increased calcium transient frequency and shortened the decay time, but the amplitude of the calcium transient was decreased (Fig. 5B and 5C). Immunostaining revealed that miR-1 and miR-541, but not NC-miRNA transfected *Dgcr8* cKO cells had well-organized sarcomere structure (Fig. 5D).

Finally, gene expression analysis revealed that, both miR-1 and miR-541 elevated the level of mature CM marker *Tnni3* to a similar level as in control CMs transfected with NC-miRNA, and significantly decreased the expression of endothelial marker *Pecam1*, which was increased in *Dgcr8* cKO cells transfected with NC-miRNA (Fig. 5E). MiR-541 upregulated cardiac progenitor marker *Nkx2–5* to a level similar in control CMs, while miR-1 did not appear to affect *Nkx2–5*. Both miR-1 and miR-541 also reduced the expression of the cell proliferation marker *Cyclin D2*, to a level even lower than NC-miRNA group (Fig. 5E).

The above results suggested that *Mesp1-Dgcr8* cKO CMs could be a useful read-out system to dissect the function of individual miRNA in early heart development, and based on this system, we uncovered that miR-541 is a novel miRNA that promotes cardiac fate and repress endothelial gene program in E9.5 embryonic heart.

### MiR-541 downregulated vascular genes and upregulated cardiac genes in cKO mouse embryonic heart cells

**Figure 6:**
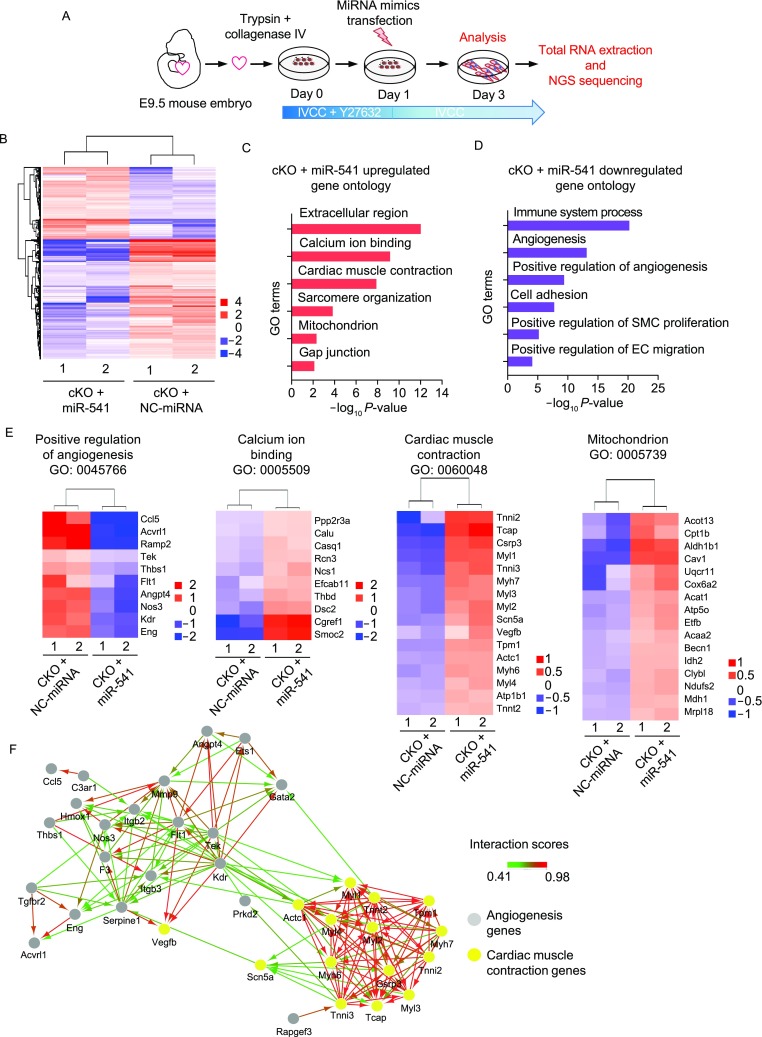
**MiR-541 selectively inhibited angiogenesis genes and promoted cardiac gene expression**. (A) Timeline of miRNA transfection into *in vitro* cultured *Dgcr8* cKO CMs followed by gene expression profiling. (B) Heatmap showing differential gene expression in E9.5 *Dgcr8* cKO CMs transfected with NC-miRNA or miR-541. Values represent normalized mean centered log_2_ of FPKM for each sample. Significantly up and downregulated genes are represented in red and blue respectively (*n* = 2). (C and D) GO analysis of genes upregulated and downregulated in *Dgcr8* cKO CMs transfected with miR-541. (E) Heatmap representation of selected genes down or upregulated in miR-541 transfected cKO CMs. The downregulated genes belonged to the GO classes of positive regulation of angiogenesis. While upregulated genes were in following GO classes: calcium ion binding, cardiac muscle contraction, and mitochondrion. Values represent normalized mean centered log2 of FPKM for each genotype. Red and blue colors representing higher and lower expressed genes respectively. (F) A Cytoscape network generated from downregulated angiogenesis genes and upregulated cardiac muscle contraction genes in miR-541 transfection group. Node color represents two types of genes, yellow and grey represents cardiac muscle contraction genes and angiogenesis genes respectively. Edge is weighted by combined interaction scores, high values correspond to red colors whereas low values to green colors

To uncover the molecular mechanism of miRNA-541 function, E9.5 heart cells from *Dgcr8* cKO embryos were cultured and transfected with miR-541-5p and NC-miRNA. 48 h after transfection, we collected total RNA from each group, performed high-throughput sequencing and analyzed changes in global gene expression (Fig. 6A). Heatmap showed that miR-541 caused significant alteration in gene expression in *Dgcr8* cKO heart cells (Fig. 6B). Compared with NC-miRNA transfected cells, 1,486 genes were downregulated in miR-541 group, they were related to angiogenesis, positive regulation of SMC proliferation, and EC migration (Fig. 6C, 6E and Table S2F). At the meantime, 1,261 genes were upregulated in miR-541 group, including genes involved in calcium ion binding, cardiac muscle contraction and mitochondrion (Fig. 6D, 6E and Table S2G). These results strongly suggested that miR-541 was at least partially responsible for the elevation of vascular genes and reduction in cardiac genes in E9.5 *Dgcr8* cKO hearts. We also performed GSEA coupled with KEGG analysis to explore pathways influenced by miR-541 (Fig. S4A). Notably, cardiac muscle contraction was overrepresented in miR-541 transfected cells, while apoptosis and p53 pathway were downregulated, which indicated that miR-541 may help to prevent apoptosis in cKO heart cells ((*P* < 0.05, FDR < 0.25; Table S4C and S4D; Fig. S4A). We also performed network analysis using Cytoscape and found that while the cardiac genes and endothelial genes formed separate networks, the links between these two major networks indicated close coordination between them to regulate heart development (Fig. 6F). MiR-541 transfection caused dramatic change in gene expression in *Dgcr8* cKO CMs, as PCA revealed that the transcriptome of miR-541 and NC-miRNA transfected cells were very far apart (Fig. S4B).

Venn diagram showed that there were 270 genes downregulated in the miR-541 transfected cKO CMs, which were also putative targets of miR-541-5p predicted by TargetScan. Moreover, 22 of them were also upregulated in *Dgcr8* cKO single CMs (Fig. S5A). In *in vitro* cultured E9.5 *Dgcr8* cKO CMs, we found that miRNA-541 significantly reduced the expression of the putative target *Ctgf* by q-PCR (Fig. S5B and S5C), while the activity of luciferase reporter bearing *Ctgf* 3′UTR was reduced by more than half upon miR-541-5p transfection (Fig. S5D). In addition, the level of CTGF protein in SVEC4-10 cell line were significantly downregulated after miR-541-5p transfection but unchanged with NC-miRNA or miR-1a-3p transfection (Fig. S5E). These results are in agreement with our hypothesis that miRNA played critical roles in vascular gene program repression to promote normal CM specification in E9.5 hearts.

### MiRNA-541 suppressed blood vessel formation and promoted CM differentiation from pluripotent stem cells

**Figure 7:**
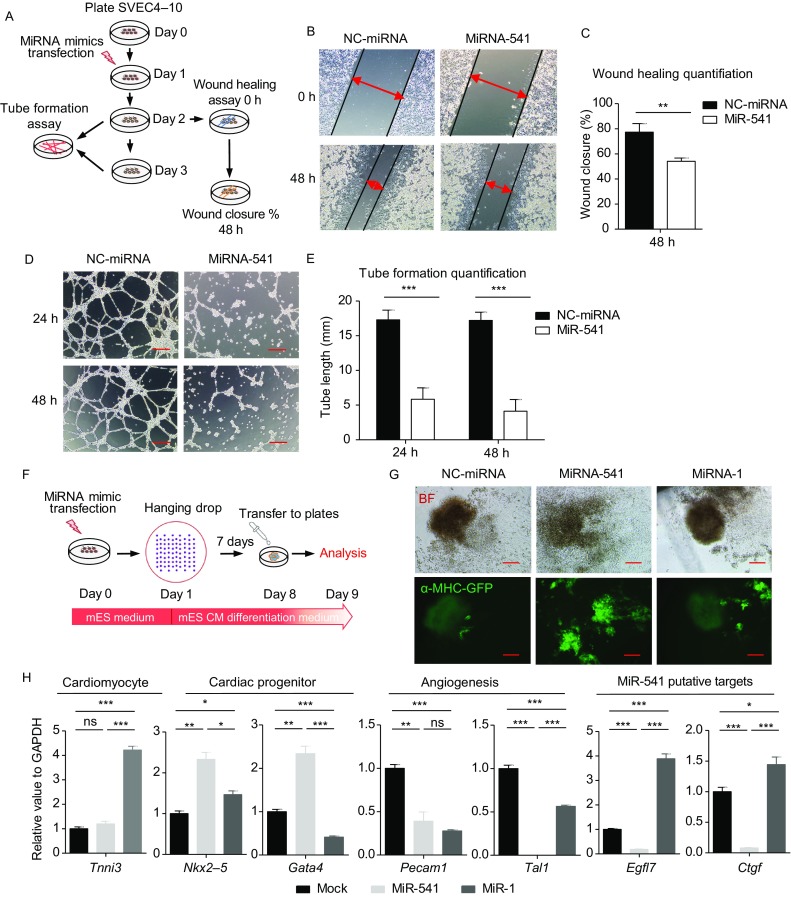
**MiR-541 disrupted blood vessel formation and promoted mESC CM differentiation**. (A) Schematic view of miR-541 transfection and endothelial function test in SVEC4–10 cells. (B) Images of scratch-wound assay of SVEC4–10 cells transfected with NC-miRNA and miR-541 at 0 and 48 h. (C) Quantification of wound closure at 48 h. Mean ± s.e.m. is shown from three biological repeats. Student’s *t* test was used: **P < 0.01. (D) Images of tube formation assay of SVEC4–10 cells transfected with NC-miRNA and miR-541 for 24 and 48 h. (E) Quantification of tube length in (D). Mean ± s.e.m. are shown from three biological repeats. Student’s *t* test was used: ***P < 0.0001. (F) Time line of miR-541 transfection in mESC and CM differentiation assay. (G) Images showing after 10 days of differentiation, *α-MHC*-GFP reporter expression in CMs derived from mESC transfected with NC-miRNA, miR-1 or miR-541. (H) Q-PCR quantification of cardiac and endothelial marker gene expression in cardiac differentiated cells derived from mESC transfected with NC miRNA, miR-1 or miR-541. Mean ± s.e.m. are shown from three biological repeats. *P < 0.05, **P < 0.01, ***P < 0.0001

Next we tested whether miR-541 can disrupt endothelial functions and facilitate CM differentiation in normal cells. To this end, we transfected NC-miRNA and miR-541 into SVEC4-10 cells which is a mouse EC line (Fig. 7A). 24 h after transfection, a scratch wound was introduced. 48 h later, the wound healing was significantly delayed in miR-541 transfected group compared to NC-miRNA transfected group (Fig. 7B and 7C). In another set of experiments, 48 h after miRNA transfection, we transferred same number of SVEC4-10 cells onto matrigel coated plate for tube formation assay. Cells in NC-miRNA group showed well-organized tubular network after 12 h, while miR-541 group showed significant defects in tube formation and reduction in total tube length (Fig. 7D and 7E). To test miR-541 function in primary endothelial tissue, we obtained E9.5 embryonic yolk sac, which was rich in blood vessels at this stage, and dissociated them into single cells and cultured *in vitro*. Transfection of miR-541 caused upregulation of CM genes such as *Tnni3*, and marked downregulation of EC gene *Pecam1* and *Tal1* (Fig. S6A and S6B). The positive control miR-1 also showed similar trend (Fig. S6A and S6B). Since miRNA-541 could also target human putative genes as predicted by TargetScan, we performed miRNA transfection and tube formation assay using ECs derived from human pluripotent stem cells (hPSCs). Similar to the results obtained with mouse ECs, miR-541 blocked tube formation of human ECs (Fig. S6C and S6E). Taken together, miR-541 severely inhibited blood vessel formation in both mouse ECs and hPSC derived ECs, which was consistent with our hypothesis.

Finally, we tested whether miR-541 may promote CM differentiation from mESCs. Undifferentiated mESCs carrying a CM specific *α-MHC*-GFP reporter were transfected with NC-miRNA, miR-541 and miR-1 respectively, then they were dissociated and CM differentiation was induced by the hanging drop method (Fig. 7F). 10 days later, *α-MHC*-GFP positive beating clusters were formed in all groups (Fig. 7G). Interestingly, q-PCR study showed that *Tnni3* increased the most in miR-1 group, while cardiac progenitor genes *Gata4* and *Nkx2-5* were higher in miR-541 group. Both miR-1 and miR-541 strongly repressed EC gene *Pecam1* and *Tal1*. Interestingly, miR-541 target genes, *Egfl7* and *Ctgf*, which were key regulators of blood vessel development, were suppressed only in miR-541 group (Fig. 7H). These results suggest that miR-541 could enhance cardiac program and repress endothelial fate during CM differentiation from pluripotent stem cells.

## Discussion

In this study, by conditional deleting microprocessor *Dgcr8* in early heart cells in mouse embryos, we uncovered that one of the miRNAs’ function is to repress vascular gene network to promote normal cardiac fate specification. We performed series of bulk and single-cell transcriptome and miRNA profiling, and for the first time, revealed miRNAs expressed in E9.5 mouse embryonic heart, which was significantly different from the adult heart. Without miRNAs, the transcriptome of early heart gained obvious vascular signature. We also established an *in vitro* culture system of E9.5 heart cells from *Dgcr8* cKO embryos and used it to dissect the function of individual miRNA. We found that miR-541, which was transiently highly expressed in E9.5 heart, acted as a strong suppressor of endothelial function and could partially rescue the defect of *Dgcr8* cKO CMs.

### Global microRNA knock-out lead to defects in E9.5 CM structure and function that correlated with significant change in the transcriptome

Tissue specific deletion of genes essential for miRNA biogenesis revealed global microRNAs are essential for organ development and the maintenance of tissue homeostasis. For example, Cardiac-specific knockout of *Dicer* by *α-MHC-Cre* lead to progressive dilated cardiomyopathy (DCM), heart failure, and premature lethality but not embryonic lethality (all mutant mice die within 4 days after birth) (Chen et al., 2008). Muscle-specific deletion of *Dgcr8* using *Mck-Cre* also resulted in DCM at 3 weeks of age, and postnatal lethality (all mutant mice died before 2 months of age and the median survival was 31 days) (Rao et al., 2009). In embryos, using CM progenitor gene *Nkx2–5* driving Cre to delete *Dicer* caused pericardial edema and poorly developed ventricular myocardium resulting in embryonic lethality at E12.5 (Zhao et al., 2007). However, miRNA loss of function study has not been performed in earlier developmental stages when the heart tube just form and undergoing dramatic transformation towards a 4-chambered heart. In current study, we performed *Dgcr8* deletion in cardiovascular progenitor cells using *Mesp1-Cre*. *Mesp1* was expressed in lateral mesoderm cells (Saga et al., 1999), which formed the cardiac crescent, then fuse to form the heart tube and primitive heart. We also observed severely dilated heart as early as E9.5, which resulted in embryo death after E10.5. *Dgcr8* cKO CMs had disrupted sarcomere structure, abnormal calcium transient and proliferation defect. Intriguingly, transcriptome analysis revealed that there was significant upregulation of angiogenesis genes in *Dgcr8* cKO heart at E9.5, and many of these genes were normally downregulated from E8.5 to E9.5 in the heart.

### Single cell RNA-seq identified significant upregulation of vascular genes in *Dgcr8* cKO CMs

Recently, single cell RNA sequencing technology has been used to dissect the cellular composition and their transcriptome during heart development and cardiac reprogramming (DeLaughter et al., 2016; Lescroart et al., 2018; Li et al., 2016; Liu et al., 2017; Zhou et al., 2016). In our single cell RNA-seq analysis of cKO and control hearts, CM, EC and other cell types can be readily separated based on lineage-specific genes compiled from previous study (Fig. 3C) (Li et al., 2016), this further proved the quality and fidelity of our experimental procedure. Our PCA graph demonstrated that the transcriptome of *Dgcr8* cKO single CMs were clearly different from that of control CMs (Fig. 3D). Moreover, angiogenesis was the top lineage related GO term of genes significantly upregulated in cKO CMs. This finding strongly supported our hypothesis that miRNA function as repressor of vascular gene network in CMs at this stage. Other over-represented GO class includes focal adhesion, glycolysis process, stress fiber etc., while significantly downregulated genes belong to the GO class of RNA binding, mitochondrion and cell cycle etc (Fig. 3F and 3G). These changes could explain the disorganized sarcomere structure, abnormal calcium transient and decreased cell proliferation in cultured cKO CMs (Fig. 1G–K). There were also some discrepancies between our single cell RNA-seq analysis of E9.5 heart and two earlier reports. In our samples, control group and *Dgcr8* cKO group contained 37% and more than 90% CMs respectively, while Li and colleagues found 70% CMs and DeLaughter and colleagues found 90% CMs in E9.5 heart (DeLaughter et al., 2016; Li et al., 2016). We think this may be partly due to different isolation method. We dissected, dissociated and manually picked single cells from the ventricular part of the E9.5 heart tube. To avoid doublets and small cell clusters, we chose visually distinct single cells. It was evident that cKO CMs had different adhesion property and dissociated much easier than control CMs which tend to cluster together. Therefore, under the same dissociation condition, more single cKO CMs were picked. While the other study used Fluidigm (integrated fluidic circuits (IFCs)) to collect dissociated single heart cells, and did not have mutant cells with altered cell adhesion property. Nevertheless, after careful statistical analysis of the RNA-seq data from our control single CMs and that from Li and DeLaughter studies, our CMs expressed high levels of CM specific marker genes and can be clustered together, which proved that they represented CM cell type in E9.5 heart. Taken together, through single cell transcriptome analysis, it was evident that the enlarged and dilated heart phenotype was the consequence of gene expression profile changes in CMs, not due to increase in EC population. Moreover, single cell analysis also helped to better identify candidate genes that may contribute to the phenotype. Thus, our study and others demonstrated that single cell RNA-seq can be a powerful tool to resolve problems associated with cell heterogeneity and advance mechanistic study (DeLaughter et al., 2016; Lescroart et al., 2018).

As Mesp1 lineage cells also form ECs and endocardium in the heart, we considered the possibility that whether defects in endocardium and ECs may contribute to the dilated heart phenotype at E9.5. We performed *in situ* hybridization of endocardium marker gene *Nfatc1* and endothelial marker *Pecam1*, respectively (Fig. S1C). *Nfatc1* was specifically expressed in the endocardium of early developing heart at E9.5, and its expression level decreases at later embryonic and neonatal stages (Zhang et al., 2017). We found that *Nfatc1* is expressed in the inner layer of E9.5 cKO embryonic hearts, and *Pecam1* could also be obviously detected in the E9.5 cKO heart, which means both endocardium and ECs were also exist in the cKO heart (Fig. S1D). *Nfatc1* signal appeared weaker in cKO hearts compared to control hearts. In cKO heart sections, the endocardial layer appeared fragile and disorganized (arrow heads), this may explain why in our single cell analysis, the cells from cKO ventricle were mostly CMs, as the proportion of endocardium cells is low in the ventricle and they may be damaged during dissociation process. In other published single cell RNA-seq study of embryonic mouse heart, the proportion of ECs was also quite low, about 10% in E9.5 ventricle. And the endocardial cell population may be even lower (DeLaughter et al., 2016; Li et al., 2016) which is in agreement with our study. Even without endocardium or ECs, as in *Mesp1Cre*; *Flk1* null E9.5 hearts, there was no dilation, and key CM marker genes were expressed normally in the myocardium (Milgrom-Hoffman et al., 2011). Therefore, it was very unlikely that endocardium and ECs which only comprised small proportion of E9.5 heart, caused the severe dilation phenotype. Taken together, as CMs comprised the majority of the ventricular cells, we think the dilated heart was mostly caused by defective CMs, not by endocardial cells.

### MicroRNA-seq of E9.5 heart uncovered miR-541 which can suppress angiogenesis and promote cardiogenesis

In this study, we also profiled microRNAs in E9.5 hearts for the first time. MiRNA-1 was the most abundant miRNA, although it only accounted for 20% of all miRNAs at this time versus 40% in adult hearts (Rao et al., 2009). Interestingly, some of the top 20 miRNAs in E9.5 heart was not highly expressed by the adult heart. We choose to investigate miRNA-541-5p, which was transient highly expressed in E9.5 heart, then decreased in later stages. MiRNA-541 was reported to reverse Ang-II induced cardiac hypertrophy by downregulation of ERK1/2 signaling (Liu et al., 2014). However, its function in early heart development was unknown. Curiously, miR-541 was enriched in E9.5 heart and downregulated afterwards. Through comparative gene expression analysis combined with miRNA target prediction, we found that 8 miR-541 putative target genes were upregulated in *Dgcr8* cKO hearts. Network analysis by STRING showed that the downregulated angiogenesis genes and upregulated cardiac genes upon miR-541 transfection formed separate regulatory modules but also had protein interactions with each other (von Mering et al., 2005), supporting the notion that there is a balance between cardiac and vascular gene program in E9.5 heart (Fig. 6F). In our experiments, miR-541 transfection disrupted scratch-would closure and tube formation of ECs (Fig. 7B and 7C). One of the target genes of miR-541 is *Ctgf* (Connective tissue growth factor), which was among the significantly upregulated angiogenesis genes identified in our single cell study.

*Ctgf* is a member of the matricellular protein family known as the CCN family, and has been shown to induce proliferation, migration, and survival of vascular ECs (Babic et al., 1999; Kubota and Takigawa, 2007; Shimo et al., 1998, 1999). Using luciferase reporter assay and q-PCR, we confirmed that *Ctgf* is as a direct target of miRNA-541 at E9.5. In addition, *Ctgf* was reported to be the target of other miRNAs such as miR-133 and miR-30 (Duisters et al., 2009), which among the top 30 highest expressed miRNAs in E9.5 heart. *Ctgf* may be one of the critical angiogenesis regulator suppressed by miRNA in the CMs. *Ctgf* was reported to promote tube formation of vascular ECs (Shimo et al., 1999), which is in agreement with our result that miR-541 severely inhibited blood vessel formation (Fig. 7D and 7E). Human CTGF had been shown to enhance tumor growth by increasing angiogenesis, and its monoclonal antibody inhibited tumor growth and metastases (Dornhöfer et al., 2006). Thus, mmu-miR-541 potentially may have clinical value in treating blood vessel hyperplasia diseases or blocking metastasis of cancer cells. Overexpression of miR-541 in ESCs promoted CM differentiation (Fig. 7G), suggesting it may be used to improve the efficiency of CM production from pluripotent stem cells and regulate myocardial remodeling in disease conditions.

**Figure 8:**
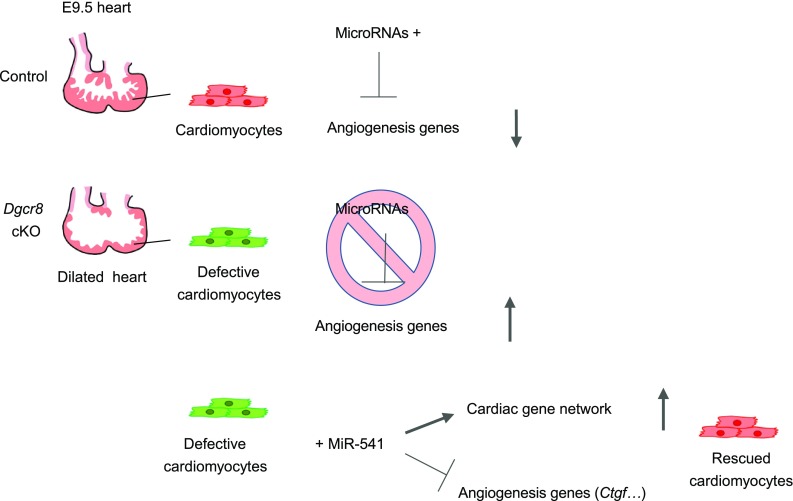
Model of miRNA in regulating gene program balance in E9.5 CMs. Knocking out global microRNA in early embryonic heart leads to severely dilation of the heart chamber and increase of angiogenesis genes in cKO CMs. MicroRNAs such as miR-541-5p function to suppress abnormal angiogenesis gene program in order to maintain normal cardiomyocyte differentiation

Based on our results, we propose a model for microRNA function during early heart development (Fig. 8). Global microRNAs are required to repress angiogenesis genes in newly formed CMs. Without microRNAs, E9.5 heart became severely dilated due to defective CMs with abnormally upregulated vascular genes. MiR-541, which was transiently highly expressed in E9.5 heart, can suppress angiogenesis gene program thus promoting cardiac gene expression. In conclusion,our study provided rich information about global miRNA expression and function during early mammalian heart development and cardiovascular progenitor cell fate decision. We propose that there is a balance between cardiac or vascular gene program during E8.5 to E9.5, a critical time window that the heart tube was transformed to a muscular organ, and miRNAs were responsible for downregulation of vascular genes and upregulation of cardiac genes. We discovered the unexpected role of miRNA-541 during this time window to suppress endothelial genes and function, which may be exploited to treat blood vessel hyperplasia or promote cardiac regeneration.

## Materials and methods

### Mouse embryos

For mouse embryo experiments, all animal protocols used in this study were approved by the IACUC (Institutional Animal Care and Use Committee) of Laboratory Animal Research Center of Tsinghua University (accredited by the AAALAC (Association for Assessment and Accreditation of Laboratory Animal Care International)). *Dgcr8* KO mice were reported previously (Wang et al., 2007). *Mesp1*^*Cre*/+^*Dgcr8*^*loxP*/+^ mice were crossed with *Dgcr8*^*loxP*/*loxP*^ or *Dgcr8*^*loxP*/*loxP*^; *ROSA26*^*mTmG*/*mTmG*^ to generate *Mesp1*^*Cre*/+^*Dgcr8*^*loxp*/*loxp*^ cKO mice. The genotype of embryos used in different series of experiments were listed in Table S6. Genomic DNA was isolated from tails of embryos. Primers for genotyping of *Dgcr8* conditional KO mice were forward 5′ ctggagtaggaatgttgatttc 3′ and reverse 5′ cctgattcacttacaacacaacc 3′. The PCR products for wide-type were 700 bp, for floxed *Dgcr8* were 900 bp and 700 bp.

### Embryo section and HE staining

Mouse E8.5 to E9.5 embryos or embryonic hearts were dissected and washed with cold PBS and fixed in paraformaldehyde overnight at 4 °C. The samples were dehydrated using automation-tissue-dehydrating machine (LEICA ASP200S) and embedded in paraffin. Then they were subjected to sectioning (5 μm thick) and H&E staining following the standard protocol.

### Immunostaining and antibodies

For immunostaining, tissue sections were blocked with 5% goat serum/PBS 0.1% Tween 20, incubated in primary antibody (cTnT 1:500 (R&D, MAB1874); PH3S10 1:500 (Cell Signaling, 9701S)) in blocking solution at 4 °C overnight. Secondary antibodies (Goat anti-rabbit Delight 549, 1:300, Thermo-fisher 35557; Goat anti-mouse Delight 488, 1:300, Thermo-fisher, 35502) in blocking buffer were applied for 1 h at room temperature. Fluorescent images were taken on a Nikon TiE inverted fluorescence microscope with 10× and 20× objectives and Element 6 software package. Confocal images were taken on Nikon A1RSi + TIRF + DMD confocal microscope.

### Isolation, culture and transfection of mouse embryonic cardiac cells

Hearts were dissected from E8.5–9.5 mouse embryos, washed in PBS without Ca^2+^, and digested using 0.04% Trypsin and 0.05% Collagenase IV. After incubation at 37 °C for 20 min (E8.5 embryonic hearts) or 30 min (E9.5 embryonic hearts), cells were gently pipetted and transferred to DMEM containing 10% fetal bovine serum for termination. Cells were plated on matrigel (1:60, BD) coated plates in the *in vitro* CMs culture medium (IVCC), with 4 ng/mL zbFGF. The *in vitro* CMs culture medium (IVCC) is composed of DMEM/F12 (Gibco) medium with 2% B27 (Gibco), 1% N2 (Gibco), 1% Glutamax (Gibco), 1% non-essential amino acids (NEAA; Gibco), 100 units/mL penicillin and 100 µg/mL streptomycin (Gibco). 50 µg/mL 2-phospho-l-ascorbic acid (Vitamin C, Sigma), 0.001% Progesterone (Sigma), 1% MEM Vitamin (Sigma, M6875), 0.1% trace element B (Corning), and 0.1% trace element C (Corning).

After 24 h culture, CMs were transfected with 50 nmol/L miRNA mimics (miRNA-1-3p, miR-541-5p and NC-miRNA (GenePharma) using Lipofectamine^®^ 2000 (Thermo Fisher) following the manufacturer’s instructions. The same transfection protocol was used for SEVC4–10, mouse Yolk sac dissociated cells, human iPSC derived ECs and mESCs.

### Transmission electron microscopy

For transmission electron microscopy, cells were cultured in 35 mm petri-dish coated with 1:60 matrigel. When cell confluence reached 70%–80%, cells were fixed in 1.25% glutaraldehyde in IVCC medium for 1 h, followed by fixation in 2.5% glutaraldehyde overnight at 4 °C. Samples embedding and section was performed by TEM facility at Tsinghua University. The TEM images were acquired using a Hitachi H-7650 TEM at 40 kV.

### Calcium transient detection and analysis

To visualize calcium transients, 10 μmol/L Fluo-4 AM (Invitrogen) was used. Time-lapse fluorescence images were recorded on a Nikon TiE inverted fluorescence microscope. Changes in Fluo-4 fluorescence (indicating fluctuation in cytosolic Ca^2+^) were recorded 100 ms per frame with a 20× objective. The fluorescence intensity change over time was quantified by Element6 software package and Image J software.

### RNA extraction, Smart-seq2 amplification and q-PCR analysis of E8.5 and E9.5 heart tube gene expression and miRNA expression

E8.5 and E9.5 wild-type and mutant heart tubes were dissected as described, and lysed thoroughly using a homogenate oscillator (Bio-Gen PRO200/PRO250, Proscientific) in 500 μL Trizol (Invitrogen). Total RNA was extracted with Trizol following manufacturer’s instruction. For mRNA q-PCR, 100 ng total RNA was reverse transcribed and amplified using Smart-seq2 (Picelli et al., 2014). Real-time q-PCR was performed using GoTaq^®^ qPCR Master Mix (Promega) in a CFX96 Real-Time System (Bio-Rad). The sample input was normalized against the Ct (critical threshold) value of the house-keeping gene *Gapdh*. Primers used were listed in Table S1.

MiRNA q-PCR was conducted following protocol described by Shi and Chiang (2005). In brief, 500 ng total RNA was polyadenylated with ATP by poly(A) polymerase (NEB, M0276S) at 37 °C for 1 h in a 20 μL reaction following the manufacturer’s directions. After phenol-chloroform extraction and ethanol precipitation, the RNAs were dissolved in nuclease free water and reverse-transcribed with 200 U SuperScript™ III Reverse Transcriptase (Invitrogen) and 0.5 μg poly(T) adapter. U6 was used as the internal reference gene for q-PCR. Primers used were listed in Table S1.

### High-throughput sequencing and data analysis

For transcriptome analysis, total RNA from E8.5 and E9.5 heart was extracted with Trizol, and 100 ng total RNA was reverse transcribed and amplified using Smart-seq2 protocol (Picelli et al., 2014) . For gene expression profiling of *in vitro* cultured E9.5 *Dgcr8* cKO heart cells transfected with miR-541-5p and NC miRNA, cells were lysed with Trizol 48 h after transfection, and 10 ng total RNA was reverse transcribed and amplified using Smart-seq2 protocol (Picelli et al., 2014). Two independent biological replicates of each group were sequenced using Illumina HiSeq2500 (Novagene, www.novogen.com; and Genewiz, https://www.genewiz.com), clean reads were mapped to mouse genome (mm9) using BWA software.

Hierarchical clustering was analyzed by the “heatmap.2” function of “gplots” R package. To see if the independent samples are separated from each other, principle component analysis was performed by SPSS. Gene ontology (GO) term enrichment was analyzed using the database for annotation, visualization, and integrated discovery (DAVID) (https://david.ncifcrf.gov).

For miRNA profiling, total RNA of E9.5 heart was extracted with Trizol, miRNA high-throughput sequencing was performed by HiSeq2500, SE50 (RIBOBIO, http://www.ribobio.com/), producing over 10 million reads from each sample. Clean reads were mapped to mouse genome (mm9) using miRDeep2 (Friedländer et al., 2008).

### Single-cell RNA sequencing

For single cell sample preparation, ventricular part of E9.5 heart tubes were dissected and digested into single cells by 0.04% trypsin combined with 0.05% collagenase IV, and then transferred into DMEM containing 10% fetal bovine serum for termination. After washed in PBS without Ca^2+^, the single cells were manually transferred into cell lysis buffer with a mouth pipette. Total RNA was reverse transcribed and amplified using Smart-seq2 protocol (Picelli et al., 2014), followed by Tn5 tagmentation and PCR enrichment to generate libraries using TruePrep DNA Library Prep Kit V2 for illumina (Vazyme, TD501-503) according to the manufacturer’s suggested protocol. The DNA libraries were sequenced on the Illumina HiSeq4000 platform with 2 × 150 bp read length per cell.

Clean reads were mapped to mouse genome (mm10) using Salmon (Patro et al., 2017). Cells with > 70% mapping rates were used for the following analysis.

PCA, Mann-Whitney-Wilcoxon rank-sum test, hierarchical clustering, volcano-plot and violin-plot were performed using custom scripts in R. Only genes expressing in > 10 cells were used for analysis. PCA and heatmap was performed on log_2_ (TPM + 1). GO term enrichment was analyzed using (DAVID) (https://david.ncifcrf.gov).

### Data availability

The RNA and microRNA high-throughput sequencing data are publicly available at the National Center for Biotechnology Information with Gene Expression Omnibus (GEO), accession number GSE96648.

### Luciferase assays

A 191 bp fragment of CTGF 3′ UTR sequence, containing putative target seed site based on the prediction of TargetScan, were cloned using the forward primer 5′ ctcgagctagtaggaaatgtggtcaaat 3′, and the reverse primer 5′ gcggccgccaactagaaaggtgcaaacatg 3′. The fragment was then ligated into the 3′ regeion of Renilla sequence in psiCheck2 vector, to generate luciferase reporter plasmids. E9.5 CMs from Dgcr8 cKO embryo were dissected and cultured *in vitro* in 96 well-plate for 16 h, before transfection of miR-541(50 nmol/L) by Lipo 2000 (Life Technologies). 6 h later, cells were transfected with reporter plasmids (80 ng/well). After 36 h, luciferase assay was performed using the Dual-Luciferase Reporter Assay System (Promega, E1910). Renilla luciferase values were normalized to firefly luciferase.

### Western blot

After miRNA transfection for 48 h, SVEC4–10 cells from each group (NC-miRNA, miR-541-5p, and miRNA-1-3p) were lysed in RIPA buffer. Cell lysates were loaded on to a 12% SDS-PAGE gel, and proteins were transferred onto nitrocellulose membranes. The membranes were blocked, followed by incubation with the primary antibody (rabbit anti-CTGF 1:1,000 (Abcam), or mouse anti-β-Actin (internal control) 1:10,000 (Abcam)) at 4 °C overnight. Membranes were then incubated with the secondary antibody (goat anti-Rabbit IgG (HRP-linked) (Jackson), 1:1,000, or goat anti-Mouse IgG (HRP-linked) (Jackson), 1:2,000) at RT. Proteins were detected using ECL solution (Millipore) and gel image system (Bio-Rad, ChemiDoc™ XRS+ System, USA).

### EC functional assays

The effects of miRNAs on mouse or human EC function were tested by vascular tube formation and scratch-wound healing assays as described in published studies (He et al., 2008; Zhang et al., 2017). In brief, for vascular tube formation assay of mouse SVEC4–10 cells (ATCC CRL-2181^TM^), 24-well plate were pre-coated with 150 μL/well Matrigel (1:1, BD Biosciences). After miRNA transfection for 24 h or 48 h, SVEC4–10 cells or human iPSC derived ECs from each group were seeded at 2 × 10^5^ cells per well and cultured at 37 °C for 12 h, 9 randomly selected fields were imaged and tube length were measured and calculated using Image J software. *n* = 3. For scratch-wound healing assay of SVEC4–10, 3 × 10^5^ cells were seeded in one well of 6-well plate, and transfected with miRNAs, 24 h after transfection, a scratch-wound were made for each group by scraping with a pipette tip. After wounding, cultures were washed twice with PBS and incubated with DMEM. Pictures were taken at 0 and 48 h after wounding and the width of the wound was measured and quantified. *n* = 3.

### Mouse ESC transfection and CM differentiation

CM differentiation from mESC R1 were performed as previously described (Ivey et al., 2008). In brief, mESCs were dissociated into single cells and resuspended in differentiation medium containing 20% FBS in DMEM. They were pipetted to form hanging drops with 1,000 cells in each 20 μL drop. 7 days later, cell aggregates in hanging drops were transferred onto gelatin coated plates. One day after plating, GFP^+^ cells could be observed. Gene expression analysis was performed on day 10.

### Construction of α-MHC-GFP reporter mouse ESC line

*α-MHC-GFP* reporter mESC R1 line was constructed using an *α-MHC-GFP* construct (Addgene plasmid 21229) (Kita-Matsuo et al., 2009) and R1 mESC followed by 400 ng/μL G418 selection for more than 2 weeks. Single *α-MHC-GFP* reporter mESC clone was verified, expanded and used for CM differentiation experiments.

### Statistical analysis

Experiments in this study were done from three biological repeats when possible. Data were presented as mean ± s.e.m. calculated using Microsoft Excel. Statistical differences were determined by unpaired two-tailed Student’s *t*-test. *P*-values < 0.05 were considered statistically significant. No statistical method was used to pre-determine sample size. No samples were excluded for any analysis.

## Electronic supplementary material

Below is the link to the electronic supplementary material.

Supplementary material 1 (PDF 8419 kb)

Supplementary material 2 (DOCX 19 kb)

Supplementary material 3 (XLSX 45 kb)

Supplementary material 4 (XLSX 746 kb)

Supplementary material 5 (DOCX 17 kb)

Supplementary material 6 (XLSX 15 kb)

Supplementary material 7 (DOCX 73 kb)

Supplementary material 8 (MOV 1485 kb)

Supplementary material 9 (MOV 2091 kb)

Supplementary material 10 (MOV 3928 kb)

Supplementary material 11 (MOV 1910 kb)

Supplementary material 12 (MOV 4663 kb)

Supplementary material 13 (MOV 3856 kb)
