# Supplementary material for: Dgcr8 deletion in the primitive heart uncovered novel microRNA regulating the balance of cardiac-vascular gene program
Source: Protein Cell. 2018 Aug 20;10(5):327–46. doi: 10.1007/s13238-018-0572-1 (PMC6468043; doi:10.1007/s13238-018-0572-1)
Supplement: Supplementary file 1 — Supplementary material 1 (PDF 8419 kb) [file 13238_2018_572_MOESM1_ESM.pdf]

Supplemental information: 6 supplemental figures

Figure S1. Phenotype of *Mesp1*$^{Cre/+}$ *; Dgcr8*$^{loxP/loxP}$ *; Rosa26*$^{mT/mG\ /+}$ embryos at different developmental stages, related to Figure 1.

Figure S2. Gene expression difference between Control and *Dgcr8* cKO embryos, related to Figure 2.

Figure S3 Quality control, cell type identification, and gene cluster analysis for single cell RNA sequencing samples, related to Figure 3.

Figure S4. Introducing MiR-541 back into *in vitro* cultured cKO cardiomyocytes lead to change in global gene expression, related to Figure 6.

Figure S5. MiRNA-541 target gene analysis, related to Figure 6.

Figure S6. MiR-541 perturbed endothelial gene expression and function, related to Figure 7.

Figure S1

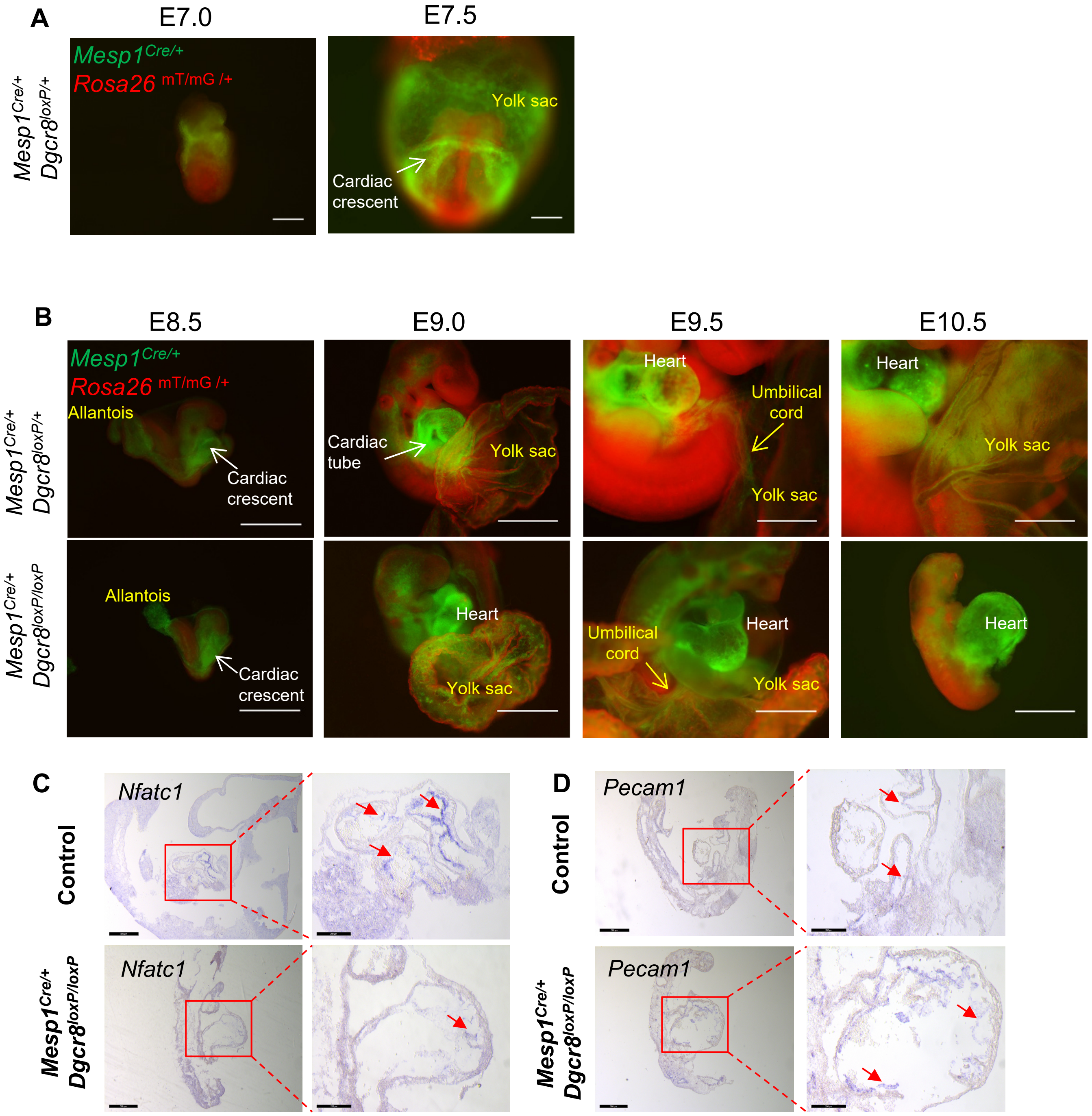

Figure S1. Phenotype of *Mesp1*$^{Cre/+}$; *Dgcr8* $^{loxP/loxP}$; *Rosa26*$^{mT/mG/+}$ embryos at different developmental stages, related to Figure 1. A, Fluorescence images of *Mesp1*$^{Cre/+}$; *Rosa26*$^{mT/mG/+}$ embryos at E7.0 and E7.5. Mesp1 lineage cells in green and other cells in red. **B,** Fluorescence images of *Mesp1*$^{Cre/+}$; *Dgcr8* $^{loxP/+}$; *Rosa26*$^{mT/mG/+}$ and *Mesp1*$^{Cre/+}$; *Dgcr8* $^{loxP/loxP}$; *Rosa26*$^{mT/mG/+}$ embryos at E8.5, E9.0, E9.5, and E10.5. Mesp1 lineage cells in green and other cells in red. Note that by E10.5, the *Dgcr8* cKO embryo already degenerated but the heart was still severely enlarged. Scale bar: 1 mm. **C-D,** *Nfatc1* (C) and *Pecam1*(D) expression in Control and cKO heart. Arrow highlights gene expression. Scale bar: 500 µm. Magnified view scale bar: 200 µm.

Figure S2

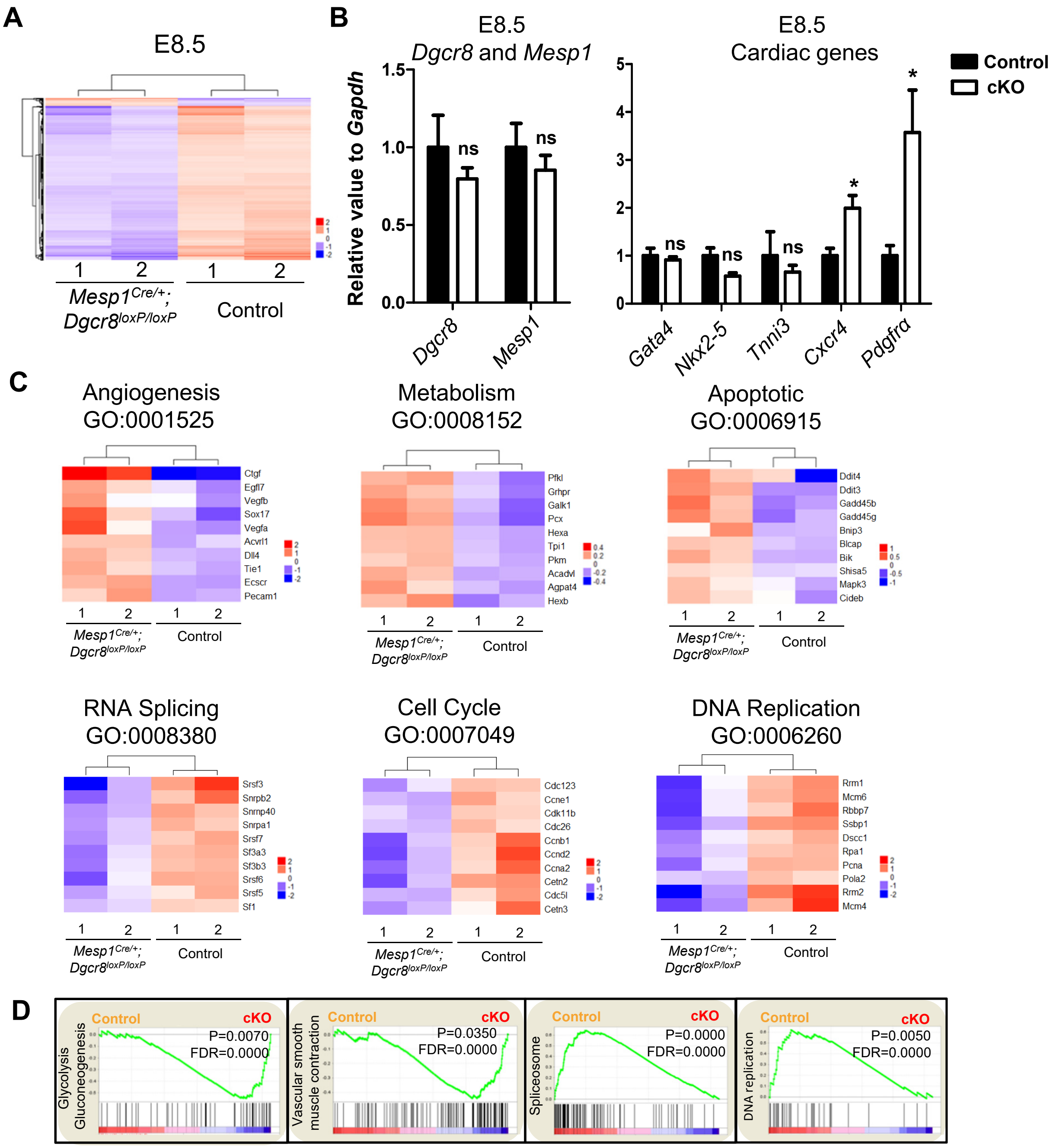

Figure S2. Gene expression difference between Control and *Dgcr8* cKO embryos, related to Figure 2. **A,** Heatmap showing difference of gene expression in E8.5 Control and *Dgcr8* cKO hearts. Values represent normalized mean centered log2 of FPKM for each sample. Significantly up and down-regulated transcripts are shown in red and blue colors respectively (n = 2). **B,** Q-PCR analysis of expression levels of *Dgcr8* and *Mesp1*, cardiac marker and progenitor cell marker genes in E8.5 Control and *Dgcr8* cKO heart tubes. Mean ± s.e.m. are shown from three biological repeats. Student's t test was used: * $P<0.05$. **C,** Heatmaps of E9.5 cKO hearts enriched genes belonging to the following GO classes: angiogenesis, metabolism, apoptotic; and Control hearts enriched genes of the GO classes: RNA splicing, cell-cycle, DNA-replication. Values represent normalized mean centered log2 of FPKM for each sample. **D,** GSEA graphs of selective pathway genes enriched in E9.5 Control and *Mesp1*$^{Cre/+}$ *Dgcr8* $^{loxP/loxP}$ embryonic hearts. $P < 0.05$, $FDR < 0.25$.

Figure S3

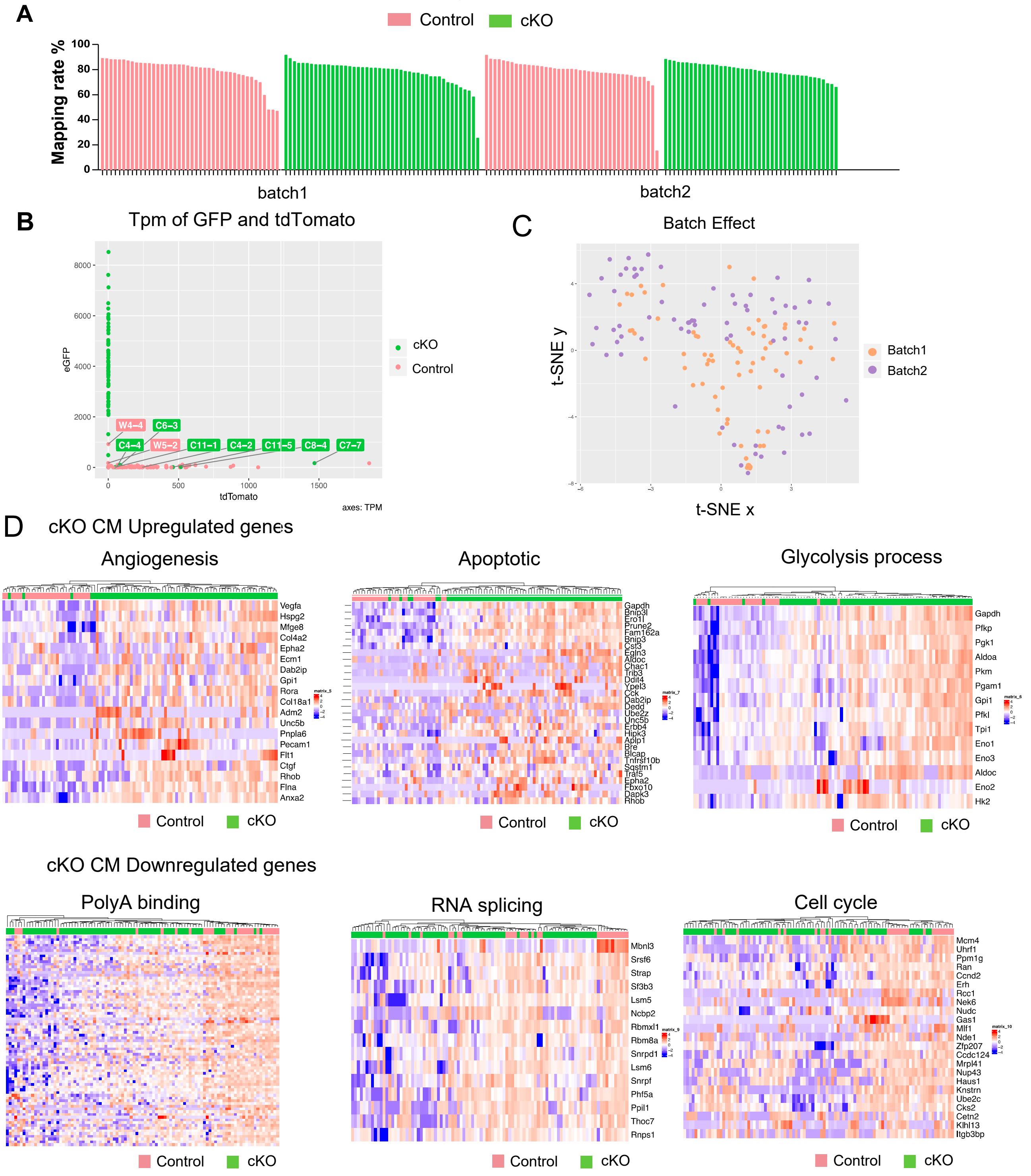

Figure S3 Quality control, cell type identification, and gene cluster analysis for single cell RNA sequencing samples, related to Figure 3. A, Bar plot of mapping rates to mouse mm10 reference genome of all single cell sample. **B,** Dot plot showing GFP and tdTomato expression level in single cell samples from Control (*Dgcr8*$^{loxp/loxp}$; *Rosa26* $^{mT/mG/+}$) and cKO (*Mesp1*$^{Cre/+}$*Dgcr8* $^{loxP/loxP}$; *Rosa26*$^{mT/mG\,/\,+}$), respectively. **C,** t-SNE Graph showing no batch effect from single cell samples in two sequencing batches (76 samples for batch1 and 76 samples for batch2). **D,** Heatmap representation of selected genes down or upregulated in cKO CMs. Values represent z-scored log2 (tpm+1) for each sample. Red and blue colors representing higher and lower expressed genes, respectively.

Figure S4

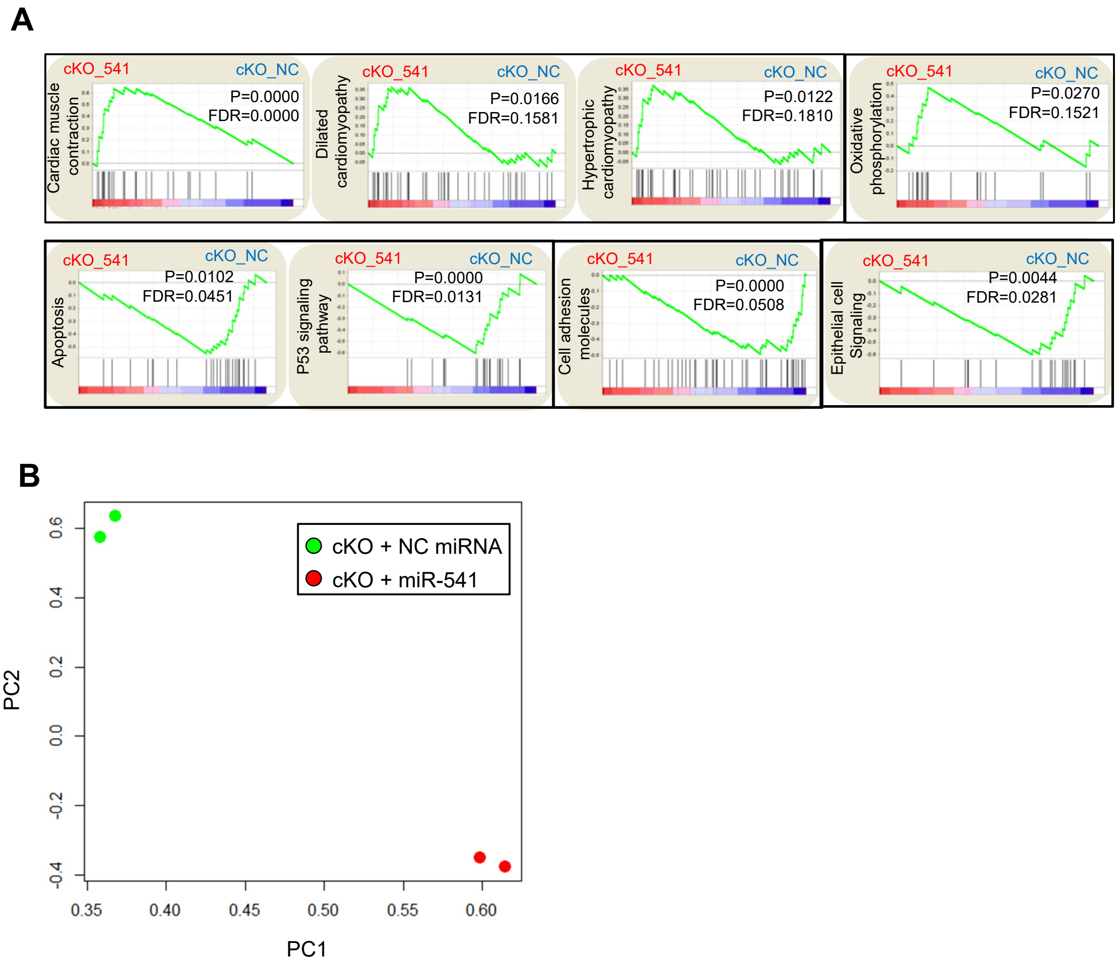

Figure S4. Introducing MiR-541 back into *in vitro* cultured cKO CMs lead to change in global gene expression, related to Figure 6. A, GSEA graph of selective pathway genes highly represented in cKO CMs transfected with miR-541(cKO_541) or NC-miRNA (cKO_NC). P < 0.05, FDR < 0.25. **B,** PCA of global gene expression in cKO cardiomyocytes transfected with miR-541 and NC-miRNA.

Figure S5

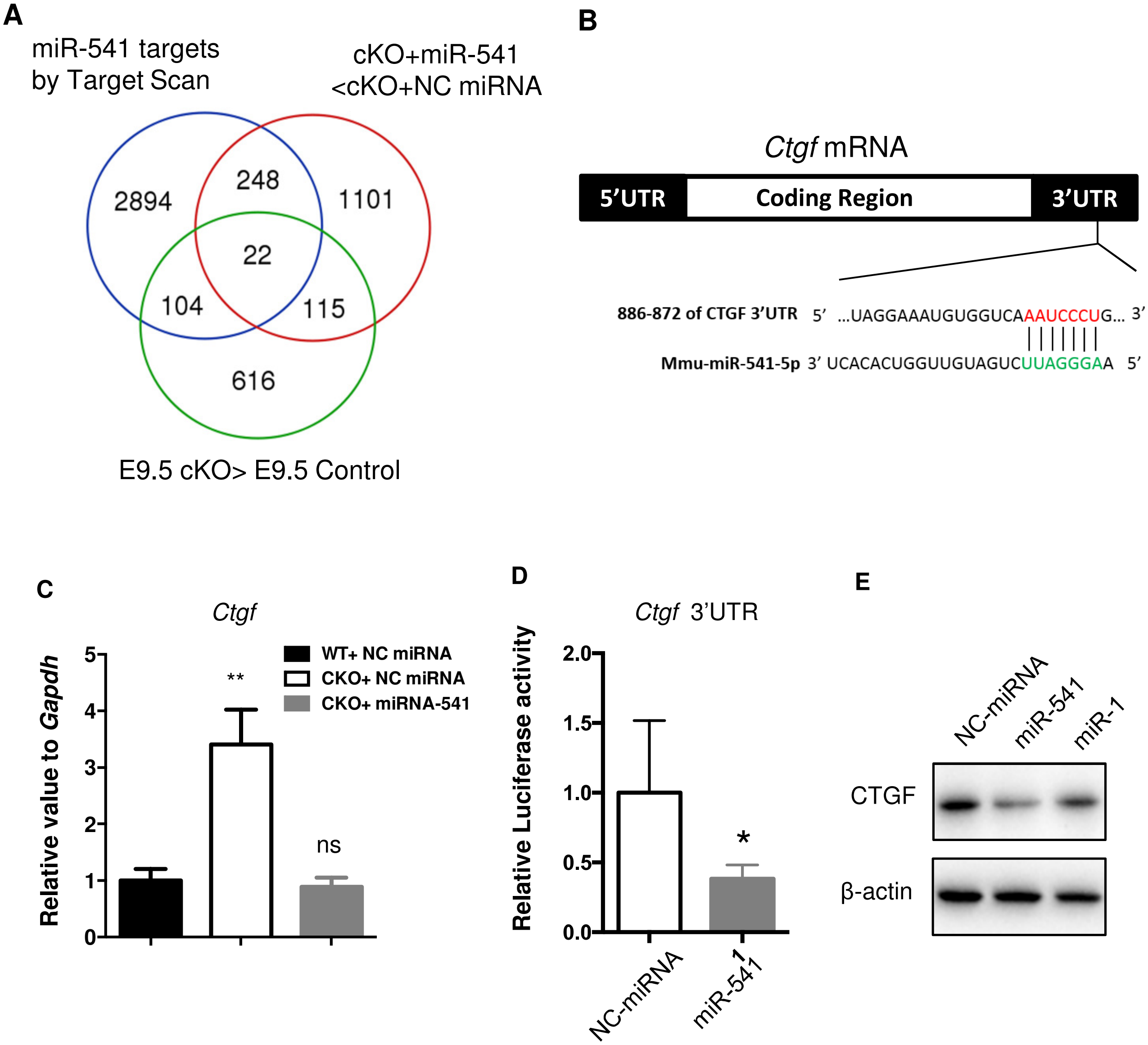

Figure S5. MiRNA-541 target gene analysis , related to Figure 6. A, Venn diagram depicting number of putative target genes of miR-541 predicted by TargetScan (blue) that overlapped with genes upregulated in E9.5 *Dgcr8* cKO heart (green), and downregulated in miR-541 transfected cKO CMs (red). **B,** A putative mmu-miR-541-5p targeting site on the 3′ UTR of *Ctgf* predicted by TargetScan. **C,** Q-PCR analysis of *Ctgf* expression in Control and *Dgcr8* cKO CMs 48 h after NC-miRNA or miR-541 transfection. Mean ± s.e.m. from three biological repeats are shown. **P<0.01. **D,** Bar graph showing luciferase activity of a reporter construct with the *Ctgf* 3′ UTR containing the miR-541 putative target site. E9.5 *Dgcr8* cKO CMs were transfected with reporter plasmids and either miR-541 mimics or control mimics. Values were normalized with blank psiCheck2 vector for each mimic group, *P<0.05. **E,** Western blot for CTGF expression in SVEC4-10 cells 48h after NC-miRNA, miR-541 and miR-1 transfection.

Figure S6

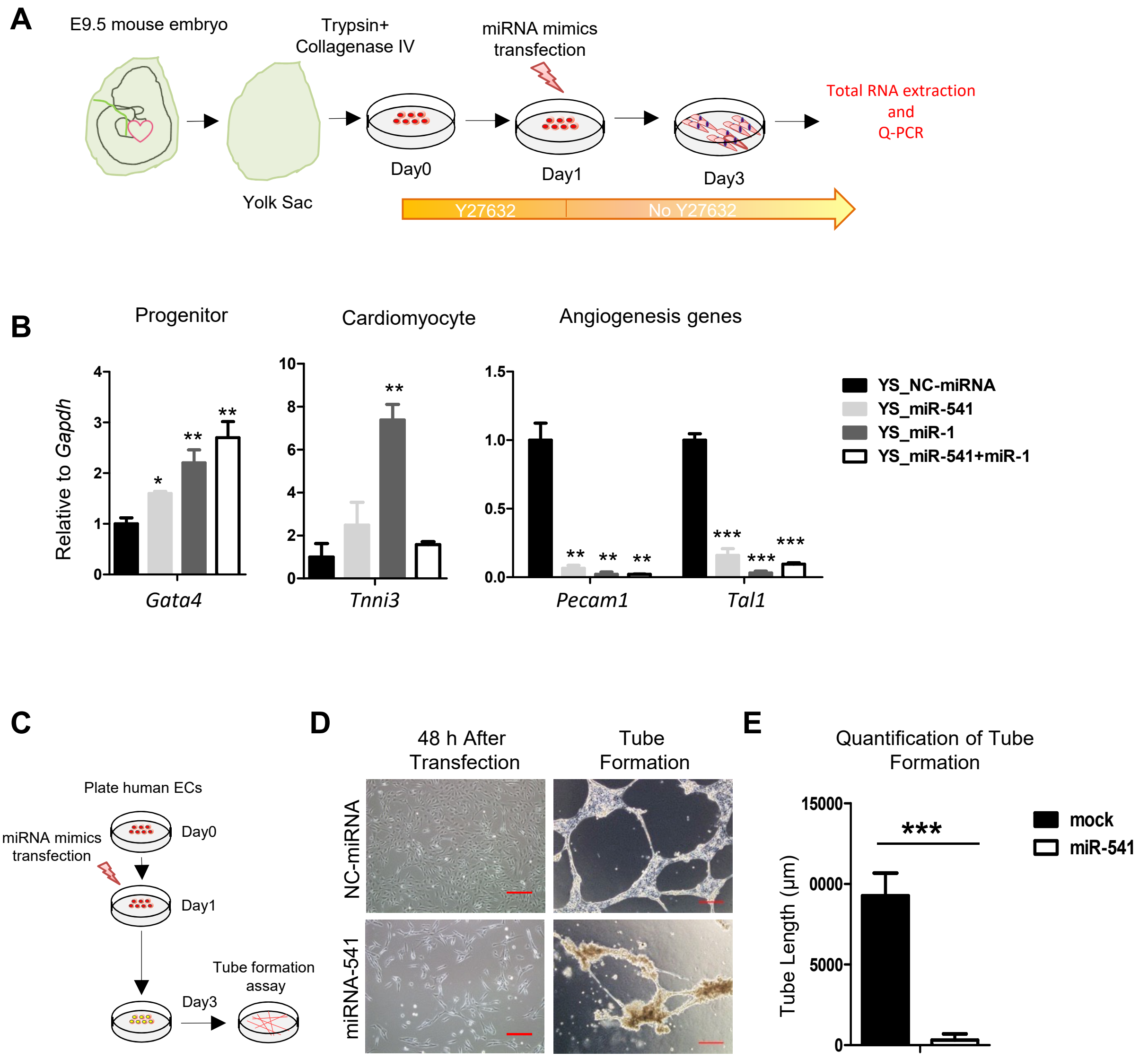

Figure S6. MiR-541 perturbed endothelial gene expression and function, related to Figure 7. A, Schematic view of miR-1 and miR-541 transfection into *in vitro* cultured Control yolk sac cells. **B,** Q-PCR analysis of gene expression in Control yolk sac cells after transfection with NC-miRNA, miR-1, or miR-541 and cultured for 48 h. Data represent mean ± s.e.m. from three biological repeats. $*P<0.05$, $**P<0.01$, $***P<0.0001$ when compared to YS_NC-miRNA using Student's unpaired t-test. **C,** Schematic view of miR-541 transfection and endothelial function analysis in human iPSC derived ECs. **D,** Images of human EC tube formation assay. ECs were differentiated from human iPSCs. Note that miR-541 also strongly inhibited human EC tube formation. **E,** Quantification of tube length in (**D**). Mean ± s.e.m. from three biological repeats are shown. $***P<0.0001$.
