# Supplementary material for: Dgcr8 deletion in the primitive heart uncovered novel microRNA regulating the balance of cardiac-vascular gene program
Source: Protein Cell. 2018 Aug 20;10(5):327–46. doi: 10.1007/s13238-018-0572-1 (PMC6468043; doi:10.1007/s13238-018-0572-1)
Supplement: Supplementary file 2 — Supplementary material 2 (DOCX 19 kb) [file 13238_2018_572_MOESM2_ESM.docx]

**Table S1. Primers for Q-PCR**

| **Gene** | **Accession** | **Forward** | **Reverse** | **Tm.** |
| --- | --- | --- | --- | --- |
| *Gapdh* | NM_008084.3 | 5’ttcaccaccatggagaaggc3’ | 5’ggcatggactgtggtcatga3’ | 60 |
| *Dgcr8* | NM_033324.2 | 5’cacggctaaagcaatcgttc3’ | 5’cctgttcccaagaagtagggt3’ | 60 |
| *Mesp1* | NM_008588.2 | 5’cccaggaaaggcaggaaatg3’ | 5’gtgccaagaccaaaggaaaagt3’ | 60 |
| *Nkx2-5* | NM_008700.2 | 5’ttcgccccccaagtgctctc3’ | 5’tccgtctcggctttgtccag3’ | 60 |
| *Gata4* | NM_008092.4 | 5’ aagacaccccaatctcgatatg3’ | 5’gatgccgttcatcttgtgatag3’ | 60 |
| *Tnni3* | NM_009406.4 | 5’ttggatgggctgggctttgaa3’ | 5’gcagagatcctcactcttcgg3’ | 60 |
| *Tbx5* | NM_011537.3 | 5’ttagggtcccagtaccagtgtg3’ | 5’tctccatgtacggcttcttatag3’ | 60 |
| *Isl1* | NM_021459.4 | 5’agcagcaacccaacgacaaaacta3’ | 5’gtatctgggagctgcgaggacat3’ | 60 |
| *Hand1* | NM_008213.2 | 5’ggtcggcaggtccttcgtgtc3’ | 5’gtgcggcgggtgtgagtgg3’ | 60 |
| *Ctgf* | NM_010217.2 | 5’ggcctcttctgcgatttcg3’ | 5’gcagcttgacccttctcgg3’ | 60 |
| *Pecam1* | NM_001032378.2 | 5’acgctggtgctctatgcaag3’ | 5’tcagttgctgcccattcatca3’ | 60 |
| *Sox17* | NM_011411 | 5’gatgcgggatacgccagtg3’ | 5’ccacctcgcctttcaccttta3’ | 60 |
| *Ecscr* | NM_001033141.1 | 5’atgcttcgagacatttctctgg3’ | 5’tgtcgtaggttgagagctgtag3’ | 60 |
| *Acvrl* | NM_001277255.1 | 5’ttggtgcagaggacggtag3’ | 5’tgctcatctcgtgaggagaaaat3’ | 60 |
| *CyclinD2* | NM_009829.3 | 5’gagtgggaactggtagtgttg3’ | 5’cgcacagagcgatgaaggt3’ | 60 |
| *Egfl7* | NM_178444.4 | 5’agcacctaccgaaccatctac3’ | 5’tgtctggcaagtatctccctg3’ | 60 |
| *Tal1* | NM_011527.3 | 5’actaggcagtgggttctttgg3’ | 5’cccggctgttggtgaagat3’ | 60 |
| *Cxcr4* | NM_009911.3 | 5’gtgaccgcctttaccccgatagc3’ | 5’tgacccccaaaaggatgaaggagt3’ | 60 |
| *Pdgfrα* | NM_001083316.2 | 5’ctggtgcctgcctcctatgac3’ | 5’cacgatcgtttctcctgccttat3’ | 60 |
| *mmu-miR-1a-3p* | MIMAT0000123 | 5’tggaatgtaaagaagtatgtat3’ | 5’gcgagcacagaattaatacgact3’ | 60 |
| *mmu-miR-541-5p* | MIMAT0003170 | 5’aagggattctgatgttggtcacact3’ | 5’gcgagcacagaattaatacgact3’ | 60 |
