# Supplementary material for: Dgcr8 deletion in the primitive heart uncovered novel microRNA regulating the balance of cardiac-vascular gene program
Source: Protein Cell. 2018 Aug 20;10(5):327–46. doi: 10.1007/s13238-018-0572-1 (PMC6468043; doi:10.1007/s13238-018-0572-1)
Supplement: Supplementary file 5 — Supplementary material 5 (DOCX 17 kb) [file 13238_2018_572_MOESM5_ESM.docx]

**Table S4. Expression of E9.5 embryonic heart enriched miRNAs (Top30), related to Figure 4**

| **MiRNA** | **Mature Read Counts**  **Sample 1** | **Mature Read Counts**  **Sample 2** |
| --- | --- | --- |
| mmu-miR-1a-1-3p | 492873 | 503921 |
| mmu-miR-1a-2-3p | 492871 | 503920 |
| mmu-miR-143-3p | 361567 | 297669 |
| mmu-miR-148a-3p | 324560 | 254949 |
| mmu-miR-127-3p | 233421 | 253401 |
| mmu-miR-21a-5p | 186562 | 160726 |
| mmu-miR-126a-3p | 143108 | 138053 |
| mmu-miR-335-3p | 128378 | 128846 |
| mmu-miR-378a-3p | 102578 | 100050 |
| mmu-miR-92a-1-3p | 100633 | 112216 |
| mmu-miR-381-3p | 87372 | 76801 |
| mmu-miR-92a-2-3p | 79713 | 90257 |
| mmu-miR-26a-5p | 78821 | 87796 |
| mmu-miR-434-5p | 63877 | 75784 |
| mmu-miR-133a-3p | 60757 | 57080 |
| mmu-miR-370-3p | 60208 | 59847 |
| mmu-miR-541-5p | 52269 | 53743 |
| mmu-miR-25-3p | 47814 | 55096 |
| mmu-miR-7a-5p | 47728 | 42979 |
| mmu-miR-30d-5p | 42326 | 40448 |
| mmu-miR-218-5p | 36815 | 35377 |
| mmu-miR-27b-3p | 36361 | 30701 |
| mmu-miR-30a-5p | 34807 | 29554 |
| mmu-miR-145a-5p | 30909 | 30005 |
| mmu-miR-379-5p | 30072 | 23491 |
| mmu-miR-30c-5p | 29651 | 29928 |
| mmu-miR-20a-5p | 28910 | 26766 |
| mmu-miR-351-5p | 25937 | 35975 |
| mmu-miR-151-3p | 24713 | 27578 |
| mmu-miR-532-5p | 23542 | 21922 |
