# Supplementary material for: Dgcr8 deletion in the primitive heart uncovered novel microRNA regulating the balance of cardiac-vascular gene program
Source: Protein Cell. 2018 Aug 20;10(5):327–46. doi: 10.1007/s13238-018-0572-1 (PMC6468043; doi:10.1007/s13238-018-0572-1)
Supplement: Supplementary file 7 — Supplementary material 7 (DOCX 73 kb) [file 13238_2018_572_MOESM7_ESM.docx]

**Table S6. Genotype of embryos used in different series of experiments**

**Table S6A. Embryo genotypes used for RNA-seq studies.**

|  | *Mesp1^Cre/+^Dgcr8^loxP/+^* ×  *Dgcr8^loxP/loxP^ Dgcr8^loxP/loxP^*; *ROSA26^mTmG/ mTmG^* | | | |
| --- | --- | --- | --- | --- |
| Genotype | *Mesp1^Cre/+^*  *Dgcr8^loxP/ loxP^*  *ROSA26^mTmG/+^* | *Mesp1^Cre/+^*  *Dgcr8^loxP/+^*  *ROSA26^mTmG/+^* | *Mesp1^+/+^*  *Dgcr8^loxP/ loxP^*  *ROSA26^mTmG/+^* | *Mesp1^+/+^*  *Dgcr8^loxP/+^*  *ROSA26^mTmG/+^* |
| Phenotype | **Dilated heart** | Normal heart,  live born and normal life span | Normal heart,  live born and normal life span | Normal heart,  live born and normal life span |
| Experiment | Bulk RNA-seq and sc RNA-seq |  | Bulk RNA-seq and sc RNA-seq |  |
| Group | Dgcr8 cKO |  | Control |  |

**Table S6B. Embryo genotypes used for immunostaining and Calcium transient studies.**

|  | *Mesp1^Cre/+^Dgcr8^loxP/+^* × *Dgcr8^loxP/loxP^* | | | |
| --- | --- | --- | --- | --- |
| Genotype | *Mesp1^Cre/+^*  *Dgcr8^loxP/ loxP^* | *Mesp1^Cre/+^*  *Dgcr8^loxP/+^* | *Mesp1^+/+^*  *Dgcr8^loxP/ loxP^* | *Mesp1^+/+^*  *Dgcr8^loxP/+^* |
| Phenotype | **Dilated heart** | Normal | Normal | Normal |
| Experiment | Immunostaining, Calcium transient assay |  | Immunostaining, Calcium transient assay |  |
| Group | Dgcr8 cKO |  | Control |  |
